# Virtual Reality–Based Cognitive Training to Prevent Cognitive Decline in Older Adults With Mild Cognitive Impairment: A Systematic Review of Randomized Controlled Trials

**DOI:** 10.1111/ggi.70586

**Published:** 2026-06-07

**Authors:** I. Made Dyanta Anwar, Junaiti Sahar, Utami Rachmawati

**Affiliations:** ^1^ Master of Nursing Program, Faculty of Nursing Universitas Indonesia Depok West Java Indonesia; ^2^ Department of Community Nursing, Faculty of Nursing Universitas Indonesia Depok West Java Indonesia

**Keywords:** cognitive training, executive function, feasibility, mild cognitive impairment, older adults, virtual reality

## Abstract

**Aim:**

To synthesize randomized controlled trial evidence on the effects, feasibility, and safety of virtual reality (VR)–based cognitive training in older adults with mild cognitive impairment (MCI) or related predementia conditions.

**Methods:**

A systematic review was conducted in accordance with PRISMA 2020 and prospectively registered in PROSPERO (CRD420261290617). Electronic searches were performed in MEDLINE (via PubMed), Scopus, ScienceDirect, and MDPI Journals, with additional verification searches in the Cochrane Central Register of Controlled Trials (CENTRAL). Eligible studies were randomized controlled trials published in English between 2020 and 2025 involving adults aged ≥ 60 years who received VR‐based cognitive or cognitive–motor training. Outcomes included cognitive and functional performance, adherence, and safety.

**Results:**

Eight randomized controlled trials were included. VR‐based interventions were associated with the maintenance or attenuation of decline in selected cognitive and functional domains, particularly executive function, memory, and visuospatial ability. Effects varied across cognitive domains, immersion levels, and intervention protocols. Adherence was generally high, and reported adverse events were infrequent and mild, most commonly dizziness or visual fatigue. No serious adverse events were reported.

**Conclusions:**

VR‐based cognitive training may represent a feasible and well‐tolerated non‐pharmacological approach for older adults with MCI. Although evidence suggests potential benefits in sustaining cognitive function, substantial heterogeneity across interventions and limited geographical diversity restrict broader generalizability. Larger multicenter trials with standardized outcome measures and longer follow‐up are needed.

## Graphical abstract

Virtual reality–based cognitive training may support the maintenance of cognitive and functional domains in older adults with mild cognitive impairment. Across eight randomized controlled trials, interventions showed generally high adherence, acceptable safety, and potential benefits for executive, memory, and visuospatial function.

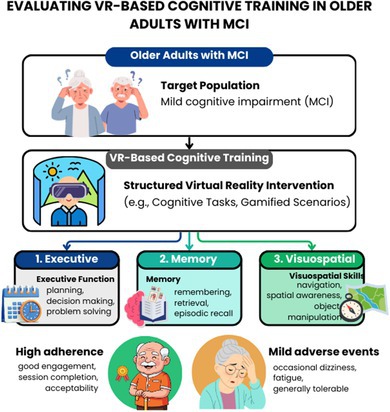

## 1 Introduction

Population aging represents a critical global demographic shift. The World Health Organization projects that the number of individuals aged 60 years and older will nearly double from 1 billion in 2020 to 2.1 billion by 2050 [1]. Similar trends are observed in many Asian countries, including Indonesia, where older adults account for 11.75% of the population [2]. In these contexts, long‐term care remains predominantly family‐based and highly gendered, with women—particularly daughters—often assuming primary caregiving roles [3]. Qualitative evidence from Indonesia highlights persistent challenges faced by family caregivers of older adults with dementia, including stigma, limited caregiver knowledge, and inadequate access to dementia‐related health services, all of which contribute to increased caregiver burden and disrupted continuity of care [4].

Within this aging population, mild cognitive impairment (MCI) has emerged as a prevalent and clinically significant condition, affecting approximately 21% of older adults in institutional settings [5]. Without effective management, MCI is associated with accelerated functional decline, increased dependency, and heightened caregiver burden [6, 7]. Although conventional non‐pharmacological interventions—such as paper‐and‐pencil memory exercises, video‐based education, and group discussions—have shown modest cognitive benefits, their long‐term effectiveness remains limited [8, 9]. These approaches are often characterized by passive engagement and low ecological validity, reducing their capacity to stimulate sustained cognitive engagement and real‐world functional relevance in older adults [9].

Virtual reality (VR) has emerged as a promising non‐pharmacological approach to cognitive training in older adults by offering interactive and ecologically valid learning environments [10]. Unlike conventional video‐based or paper‐based interventions, VR enables users to actively engage with simulated three‐dimensional settings that mirror activities of daily living, thereby promoting task‐relevant cognitive stimulation [11]. This active engagement supports situated learning processes that are particularly relevant for episodic memory and executive functioning in later life. In addition, VR‐based interventions provide multisensory feedback that engages visuospatial and executive neural networks, potentially enhancing neuroplasticity and cognitive resilience in individuals with MCI [12].

Despite its potential benefits, the integration of VR into geriatric cognitive care is not without challenges. Cybersickness, commonly manifested as dizziness, nausea, or visual discomfort, remains a primary concern, particularly among older adults who may already experience age‐related vestibular decline [13, 14]. Recent work also suggests that sensorimotor mismatches alone—when not accompanied by strong visual–vestibular conflict and when VR is delivered in seated, short‐duration tasks—may have a limited impact on sickness outcomes [15]. Physical discomfort related to headset weight and prolonged use has also been reported [16, 17]. Importantly, emerging evidence suggests that such adverse effects can be mitigated through carefully designed protocols, including shorter session durations, seated training, and optimized display performance.

Although growing evidence supports the cognitive benefits of VR‐based interventions, uncertainties remain regarding their feasibility, tolerability, and safety in older adults with MCI. Therefore, this systematic review aims to synthesize randomized controlled trial evidence on the effectiveness, adherence, and safety of VR‐based cognitive training to inform geriatric clinical practice.

## 2 Methods

### 2.1 Systematic Literature Review

This study employed a systematic literature review design conducted in accordance with the 2020 Preferred Reporting Items for Systematic Reviews and Meta‐Analyses (PRISMA) guidelines [18]. The review process comprised systematic database searches, study screening, eligibility assessment, and data extraction. These stages were undertaken independently by two reviewers to enhance methodological rigor and minimize potential bias. This systematic review was prospectively registered in the PROSPERO database (CRD420261290617).

### 2.2 Search Strategy

A systematic literature search was conducted across major bibliographic databases (MEDLINE (via PubMed) and Scopus), as well as specific publisher platforms (ScienceDirect and MDPI Journals). Furthermore, to ensure comprehensive coverage of randomized controlled trials, additional verification searches were conducted in the Cochrane Central Register of Controlled Trials (CENTRAL) to identify additional randomized controlled trials. The search covered studies published between 1 January 2020 and 31 March 2025. The search was initially conducted in March 2025 and rerun on 6 March 2026 using the same predefined date limits to verify study identification during the revision process. The search strategy combined controlled vocabulary (e.g., MeSH terms) and free‐text keywords related to mild cognitive impairment, virtual reality, and randomized controlled trials. Boolean operators (AND, OR) were used to combine search terms across concepts, and the search strategy was adapted for each database according to its indexing system.

All retrieved records were exported into reference management software, and duplicate records were removed prior to screening. In addition to database searches, backward citation tracking of included studies and relevant reviews was performed, and forward citation searches were conducted using Scopus to identify additional eligible trials. The full database‐specific search strategies are provided in the Supporting Information.

### 2.3 Eligibility Criteria

Eligibility criteria were defined using the PICO framework. The population comprised adults aged ≥ 60 years with mild cognitive impairment (MCI) or related predementia conditions, confirmed using standardized cognitive assessments (e.g., MMSE, MoCA, or equivalent validated instruments). Eligible interventions included VR‐based cognitive or memory training programs encompassing immersive, semi‐immersive, and non‐immersive systems, provided that a clearly defined cognitive training component was present. Comparator conditions included non‐VR control conditions such as usual care, conventional cognitive training, health education, physical exercise without VR, or waitlist/no‐intervention controls.

Primary outcomes of interest were changes in global or domain‐specific cognitive performance (e.g., memory, executive function, attention, and visuospatial ability). Secondary outcomes included intervention adherence and safety outcomes, including reported adverse events such as cybersickness or discomfort.

Only randomized controlled trials published in English between 1 January 2020 and 31 March 2025 were included. Studies involving diagnosed dementia, Alzheimer's disease, severe cognitive impairment, cognitive impairment secondary to neurological conditions (e.g., stroke, Parkinson's disease, or traumatic brain injury), or VR interventions focused exclusively on physical rehabilitation without cognitive components were excluded.

### 2.4 Study Selection and Data Extraction

Two reviewers independently screened titles and abstracts to assess eligibility based on the predefined inclusion criteria. Full‐text articles were subsequently reviewed to confirm study eligibility. Disagreements were resolved through discussion, with consultation of a third reviewer when necessary. Data were extracted using a standardized extraction form and included study characteristics (authors, year, country, study design), participant characteristics, intervention details (type of VR system, immersion level, duration, and frequency), comparator conditions, outcome measures, and key findings.

### 2.5 Quality Appraisal and Risk of Bias Assessment

The methodological quality of included studies was assessed using the Joanna Briggs Institute (JBI) Critical Appraisal Checklist for Randomized Controlled Trials. This tool evaluates key domains including randomization procedures, allocation concealment, baseline comparability, blinding, reliability of outcome measures, completeness of follow‐up, and appropriateness of statistical analyses. Each criterion was rated as “Yes,” “No,” “Unclear,” or “Not Applicable.”

Given the interactive nature of VR‐based interventions, blinding of participants and personnel was not feasible in several studies and was therefore considered within the context of intervention characteristics rather than as an inherent methodological flaw. Overall appraisal results are presented in Appendix 3 (Supporting Information).

### 2.6 Data Synthesis

Due to substantial heterogeneity in study designs, VR intervention characteristics (e.g., immersion level, content, and duration), and outcome measures, a meta‐analysis was not undertaken. Instead, a narrative synthesis was performed to systematically compare findings across studies. Synthesized domains included cognitive outcomes, intervention adherence, and reported safety or tolerability issues, particularly cybersickness and physical discomfort. This approach enabled structured comparison of intervention effects while accounting for clinical and methodological variability among the included trials.

### 2.7 Use of Generative AI in Manuscript Preparation

During manuscript preparation, ChatGPT (OpenAI) was used solely to assist with language refinement, structural organization, and clarity of presentation. The tool was not used to generate original data, perform analyses, determine study eligibility, extract data, assess risk of bias, or make scientific conclusions. All outputs were critically reviewed, verified, and edited by the authors, who take full responsibility for the content of the manuscript.

## 3 Results

### 3.1 Study Selection

**FIGURE 1:**
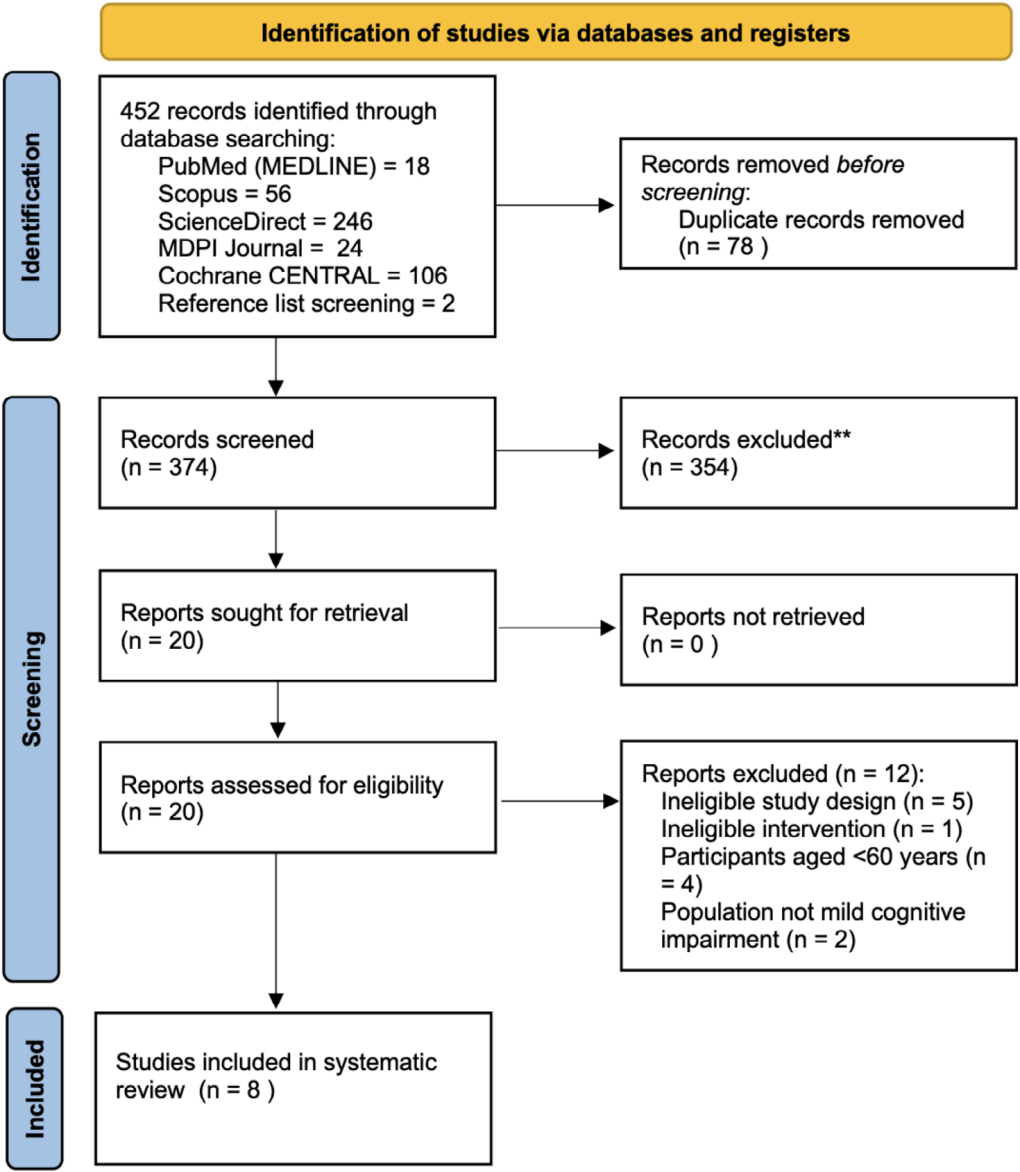
PRISMA flow chart.

The database search identified 452 records across the selected databases and additional sources. After removing 78 duplicate records, 374 articles remained for title and abstract screening. Following this stage, 354 records were excluded for not meeting the eligibility criteria. Twenty full‐text articles were assessed, of which 12 were excluded due to ineligible study design (*n* = 5), inappropriate intervention (*n* = 1), age below 60 years (*n* = 4), or non‐MCI populations (*n* = 2). Ultimately, eight randomized controlled trials met the inclusion criteria and were included in the final synthesis. The study selection process is summarized in Figure 1 (PRISMA flow diagram).

### 3.2 Study Characteristics

The eight included randomized controlled trials were published between 2020 and 2025 and conducted across five regions: South Korea (*n* = 3), Taiwan (*n* = 2), China (*n* = 1), Hong Kong (*n* = 1), and Turkey (*n* = 1). Across all studies, a total of 611 participants were included, with individual sample sizes ranging from 21 to 293 participants. Participants consisted primarily of older adults with mild cognitive impairment (MCI) or related predementia conditions, including subjective cognitive decline and cognitive frailty, as defined by study‐specific diagnostic criteria. Study settings included community, outpatient, long‐term care, and nursing home contexts. Detailed intervention characteristics and outcome measures are summarized in Table 1.

**TABLE 1:** Characteristics of included randomized controlled trials (2020–2025).

No	Author (Year, Country)	Sample (I/C)	Participants	Intervention	Immersion level	Control	Intervention period	Total sessions	Reported adverse events	Main outcomes	Key findings
1	Torpil et al. [19]	30/31	Older adults with MCI	VR‐based cognitive rehabilitation using Microsoft Kinect games	Non‐immersive	Conventional cognitive rehabilitation (CR) intervention	45 min 2×/week 12 weeks	24	None reported	The LOTCA‐G	Significant improvements in visuospatial, visuomotor, attention, and thinking domains.
2	Kang et al. [12]	23/18	Older adults with subjective cognitive decline or MCI	Fully Immersive Virtual Reality Cognitive Training	Fully immersive	Routine care only (e.g., standard pharmacotherapy).	20–30 min 2×/week 4 weeks	8	Dizziness, nausea, oculomotor disturbance, disorientation	RCFT, SVLT, TMT‐A/B, rsfMRI	Significant improvements in visuospatial function and mood, with changes in functional brain connectivity.
3	Liao et al. [20]	18/16	Community‐dwelling older adults with MCI	VR‐based physical and cognitive training (simulating IADL tasks like cooking and shopping)	Fully Immersive	Combined physical and cognitive training (CPC)	60 min 3×/week 12 weeks	36	None reported	MoCA, EXIT‐25, CVVLT, PFC activation	Significant improvements in global cognition and delayed verbal recall in the VR group.
4	Zheng et al. [21]	33/33	Older adults with cognitive frailty and ADL limitations	VR‐Based Activities of Daily Living (ADL) Rehabilitation Training	Fully immersive	Usual nursing care; maintained regular lifestyle in nursing home.	45 min 2×/week 12 weeks	24	None reported	FIM, BI, IADL, MMSE, GDS‐15	Significant improvements in cognitive function, activities of daily living, and quality‐of‐life measures.
5	Chiu et al. [22]	30/30	Older adults with cognitive impairment in LTCFs	Virtual Reality Cognitive Training Intervention (VRCTI)	Fully immersive	Routine long‐term care without additional training.	60 min 1×/week 8 weeks	8	None reported	CASI, MMSE, CDT‐D, QoL	Significant improvements in cognitive function and quality of life, with high completion rates.
6	Kwan et al. [23]	146/147	Community‐dwelling older adults with cognitive frailty	Virtual Reality Motor‐Cognitive Training (VRMCT)	Fully immersive	Usual community care; intervention provided after follow‐up period.	60 min (30 min VR) 2×/week 8 weeks	16	Vertigo, headache, difficulty focusing, eyestrain	MoCA, SCWT, TMT‐B, gait	Improvements in global cognition, gait, and frailty outcomes, with low rates of VR‐related discomfort (0.7%–3%).
7	Park et al. [24]	10/11	Patients with amnestic MCI	Culture‐Based Virtual Reality (VR) Training Program	Non‐immersive	Conventional cognitive rehabilitation (e.g., puzzles, cards, maze tasks).	30 min 5×/week 6 weeks	30	Not reported	K‐MMSE, SNSB‐D, K‐CWST	No statistically significant between‐group differences observed in cognitive outcomes.
8	Park et al. [25]	18/17	Older adults with MCI	Virtual Reality‐Based Cognitive–Motor Rehabilitation (VRCMR)	Fully immersive	Continued normal daily activities throughout the study.	30 min 2×/week 12 weeks	24	Fatigue, dizziness	MoCA, TMT‐A/B, DST	Significant improvements in cognitive and motivation‐related outcomes, with high adherence reported.

Abbreviations: ADL, activities of daily living; BI, barthel index; CASI, cognitive abilities screening instrument; CDT/CDT‐D, clock drawing test/clock drawing test–dementia version; CVVLT, Chinese version of the verbal learning test; DST, digit span test; SCWT, stroop color–word test; FIM, functional independence measure; gait, gait speed test; GDS‐15, geriatric depression scale (15‐item version); IADL, instrumental activities of daily living; K‐CWST, Korean color–word stroop test; K‐MMSE, Korean mini–mental state Examination; LOTCA‐G, Loewenstein occupational therapy cognitive assessment–geriatric; MMSE, mini–mental state examination; MoCA, montreal cognitive assessment; PFC, prefrontal cortex; QoL, quality of life; RCFT, Rey–Osterrieth complex figure test; rsfMRI, resting‐state functional magnetic resonance imaging; SNSB‐D, Seoul neuropsychological screening battery–dementia version; SVLT, seoul verbal learning test; TMT‐A/B, trail making test parts A and B.

### 3.3 Intervention Characteristics

All included studies implemented VR‐based cognitive or cognitive–motor training interventions, with variations in immersion level, training duration, and intervention content (Table 1). Five trials employed fully immersive VR systems using head‐mounted displays, while three studies used non‐immersive platforms, including screen‐based or projection‐based environments.

Intervention duration ranged from 4 to 12 weeks, with training frequencies varying between 1 and 5 sessions per week. Session length ranged from 20 to 60 min, resulting in total session counts between 8 and 36 sessions across studies.

Interventions targeted cognitive domains such as memory, attention, and executive function, often through simulated activities of daily living. Two studies incorporated combined cognitive–motor training. Control conditions across trials included usual care, conventional cognitive rehabilitation, health education, physical exercise without VR, or delayed intervention controls.

### 3.4 Cognitive and Functional Outcomes

Cognitive and functional outcomes were reported across all eight included trials, although outcome domains and assessment instruments varied substantially (Table 1). Overall, six of the eight trials reported statistically significant improvements in at least one cognitive or functional domain following VR‐based interventions compared with control conditions. Global cognitive performance was assessed using standardized instruments such as the Mini–Mental State Examination (MMSE) and Montreal Cognitive Assessment (MoCA), with four trials reporting significant post‐intervention improvements.

Executive function and attention outcomes, measured using tools including the Trail Making Test and Stroop‐based assessments, improved significantly in three studies. Memory‐related outcomes, including verbal learning and working memory, showed mixed findings, with several studies reporting significant improvements while others observed no statistically significant between‐group differences.

Functional outcomes, including activities of daily living, gait, and balance, were evaluated in four studies, three of which reported significant improvements. Two trials also reported improvements in quality of life and emotional well‐being.

### 3.5 Adherence and Safety

Adherence to VR‐based interventions was generally high across the included trials. Six studies reported attendance or completion rates exceeding 80%, indicating good feasibility and acceptability among older adults. Safety outcomes were explicitly reported in five studies. Reported adverse events were predominantly mild and transient, including dizziness, visual fatigue, headache, eyestrain, nausea, or fatigue. The proportion of participants experiencing VR‐related discomfort ranged from 0.7% to 3% in studies reporting quantitative safety data. No severe adverse events or serious cybersickness were reported. Three studies did not provide explicit safety reporting.

### 3.6 Quality Appraisal Results

Quality appraisal using the JBI Critical Appraisal Checklist indicated moderate overall methodological quality (Appendix 3 (Supporting Information)). All studies reported clear eligibility criteria and randomization procedures, although allocation concealment and blinding were inconsistently reported. Outcome measures were generally valid, dropout rates were low, and three trials did not explicitly report intention‐to‐treat analyses. Overall, limitations were primarily related to blinding and sample size variability.

## 4 Discussion

This systematic review synthesizes evidence from eight randomized controlled trials examining the effects of virtual reality (VR)–based interventions on cognitive and functional outcomes in older adults with mild cognitive impairment and related conditions. Overall, VR interventions were associated with improvements in selected cognitive domains, particularly executive function, memory, and visuospatial abilities, while adherence rates were generally high and adverse events were infrequently reported. However, outcomes varied across studies, reflecting heterogeneity in VR immersion levels, intervention duration, and outcome measures. Together, these findings suggest that VR may represent a feasible non‐pharmacological approach to support cognitive training in older adults, although the strength and consistency of effects differ across cognitive domains and intervention designs.

Improvements were most consistently reported in executive function, memory, and visuospatial abilities, particularly in interventions employing immersive and task‐oriented VR environments. These domains are closely related to goal‐directed behavior and spatial navigation, which are actively engaged when participants interact with simulated activities of daily living rather than passively receiving information. Prior studies suggest that interactive VR tasks may support executive control by requiring concurrent cognitive processing and decision‐making within dynamic environments [10, 26]. Memory‐related benefits have similarly been attributed to active exploration and navigation within virtual spaces, which may enhance encoding and retrieval processes in older adults [27]. Visuospatial outcomes appeared more frequently in studies employing fully immersive systems, where embodied interaction and environmental control are maximized, potentially enhancing the sense of presence and task engagement [28]. However, evidence also indicates that visuospatial improvement is not solely determined by visual immersion. Active navigation and meaningful interaction with the environment—rather than passive observation—may play a critical role, even in non‐immersive platforms [29]. Differences in immersion level may therefore influence which cognitive domains are most strongly engaged, with more immersive systems favoring executive and visuospatial processing and less immersive platforms supporting repetitive attention‐ and memory‐based tasks [10, 26, 28].

Feasibility and tolerability emerged as important strengths. Most trials reported high adherence rates (> 80%) and infrequent, predominantly mild adverse events (e.g., transient dizziness). These findings align with prior research suggesting that VR interventions are highly acceptable when employing user‐friendly designs, short session durations, and gradual exposure protocols [15, 30]. While cybersickness remains a relevant concern in geriatric populations, its low incidence in our review likely reflects the use of seated, task‐oriented environments. Nonetheless, the absence of standardized VR‐discomfort measurement tools in the included studies warrants cautious interpretation [31].

An additional observation from this review is the geographical concentration of the available evidence. Seven of the eight included trials were conducted in East Asia (South Korea, China, Taiwan, and Hong Kong), with the remaining trial conducted in Turkey. This pattern is consistent with recent reviews showing that a large proportion of VR‐based cognitive intervention studies originate from institutions in these regions [10, 32]. The rapid integration of digital health technologies within geriatric rehabilitation programs in East Asian healthcare systems may contribute to this trend [10, 32]. In addition, several trials reported national or institutional support for digital health innovation, which may facilitate the development of VR‐based interventions for aging populations [12, 23, 27]. Some interventions were culturally tailored, which may further limit transferability across settings [23, 29]. Nevertheless, the limited geographical diversity of the current evidence base may restrict the generalizability of findings across different healthcare systems and sociocultural contexts. Future multicenter trials conducted across diverse regions are therefore needed to strengthen the global evidence base for VR‐based cognitive interventions in older adults [30].

Another methodological challenge identified across the included trials concerns the validity of control conditions and the difficulty of implementing blinding in VR‐based interventions. Because VR training involves highly interactive and visually distinctive environments, participants and therapists are typically aware of group allocation, making full participant blinding difficult to achieve in most trials. This limitation is common in behavioral and technology‐assisted interventions and may introduce performance and expectation bias [10, 21]. Several studies attempted to address this limitation through the use of standardized control conditions such as conventional cognitive training, usual care, or delayed intervention controls [23, 29]. However, differences in engagement levels between VR and non‐VR activities may still influence outcomes, particularly when immersive environments increase motivation or novelty effects during training [15, 33]. When participant blinding is not feasible, assessor blinding becomes particularly important to reduce detection bias in cognitive outcome assessment [19, 23]. Future trials should therefore prioritize assessor‐blinded designs, active control groups with comparable cognitive tasks, and clearly standardized intervention protocols to improve internal validity and interpretability of VR‐based cognitive training effects [10, 21].

Clinically, VR offers ecologically valid, task‐oriented cognitive training by safely simulating activities of daily living. This approach is particularly valuable for older adults with mobility limitations or restricted environmental access. Integrating VR into community or long‐term care settings may complement conventional interventions by providing structured, repeatable cognitive‐functional stimulation while maintaining safety and supervision [31, 33].

Several limitations of this review should be acknowledged. First, the included trials exhibited substantial heterogeneity in VR systems, intervention content, duration, and outcome measures, which precluded quantitative synthesis. Second, several studies were limited by small sample sizes and relatively short intervention periods, potentially restricting the assessment of long‐term effects. Third, the predominance of studies from East Asia may limit the generalizability of findings to other healthcare systems and sociocultural contexts. Fourth, variability in comparator rigor and incomplete blinding may have influenced effect estimates.

## 5 Conclusion

This systematic review indicates that virtual reality–based interventions are associated with the maintenance or attenuation of decline in selected cognitive and functional domains in older adults with mild cognitive impairment, with generally high adherence and acceptable safety profiles. While cognitive effects varied across domains and intervention designs, immersive and task‐oriented approaches appeared particularly relevant for executive and visuospatial engagement. Importantly, feasibility and tolerability emerged as key strengths, supporting the potential integration of VR into geriatric and nursing practice. However, the geographical concentration of the current evidence base and substantial intervention heterogeneity limit broader generalizability. Future research should prioritize larger, well‐designed multicenter trials with standardized outcome measures, optimized training schedules, and longer follow‐up to clarify the role of VR in sustaining cognitive function in aging populations.

## Ethics Statement

The authors have nothing to report. This study is a systematic review of previously published studies and did not involve direct participation of human subjects or the collection of primary data.

## Conflicts of Interest

The authors declare no conflicts of interest.

## Supporting information


**Appendix 1:** PICO worksheet and search strategy.

**Appendix 2:** Supplementary Table—Literature search results; PRISMA flow; Data extraction.

**Appendix 3:** The JBI critical appraisal tool for RCTs Article 1.
The JBI critical appraisal tool for RCTs Article 2.
The JBI critical appraisal tool for RCTs Article 3.
The JBI critical appraisal tool for RCTs Article 4.
The JBI critical appraisal tool for RCTs Article 5.
The JBI critical appraisal tool for RCTs Article 6.
The JBI critical appraisal tool for RCTs Article 7.
The JBI critical appraisal tool for RCTs Article 8.

## Data Availability

Data sharing not applicable to this article as no datasets were generated or analysed during the current study.
