# Supplementary material for: Virtual Reality–Based Cognitive Training to Prevent Cognitive Decline in Older Adults With Mild Cognitive Impairment: A Systematic Review of Randomized Controlled Trials
Source: Geriatr Gerontol Int. 2026 Jun 7;26(6):e70586. doi: 10.1111/ggi.70586 (PMC13243133; doi:10.1111/ggi.70586)
Supplement: Supplementary file 1 — Appendix 1: PICO worksheet and search strategy. [file GGI-26-0-s001.docx]

# PICO Worksheet and Search Strategy

1. **Define your question using PICO or other formats by identifying: Problem, Intrvention, Comparison Group and Outcomes**

| **Patient/Problem Patient** | Older adults aged ≥60 years diagnosed with mild cognitive impairment (MCI) or predementia, |
| --- | --- |
| **Intervention** | Virtual reality–based cognitive training (fully immersive, semi-immersive, or non-immersive systems) with a clearly defined cognitive training component. |
| **Comparison** | Non-VR control conditions (e.g., usual care, conventional cognitive training, health education, physical exercise without VR, or waitlist). |
| **Outcome** | Primary: Changes in global or domain-specific cognitive performance (e.g., memory, executive function, attention, visuospatial ability). Secondary: Adherence and reported adverse events. |

1. **Write your question**

“In older adults aged ≥60 years with mild cognitive impairment (MCI) or predementia, does virtual reality–based cognitive training, compared with non-VR control conditions, maintain or improve cognitive function?”

1. **Type of question/problem**

**✔️Therapy/prevention**

☐ Diagnosis

☐ Etiology

☐ Prognosis

1. **Type of Research:**

☐ **Meta-analysis**

**✔️Systematic review**

**✔️Randomized Controlled Trial**

☐ Cohort study

☐ Case control study

☐ Case series or case report

☐ Editorials, Letters, Opinions

☐ Animal Research

☐ Vitro/Lab research

1. **List main topics/concepts and alternate term from your PICO question that can be used for your search**

| P | I | C | O |
| --- | --- | --- | --- |
| “mild cognitive impairment” OR MCI | “virtual reality” OR VR | usual care | cognitive function |
| “amnestic MCI” | immersive virtual reality | conventional cognitive training | cognitive performance |
| “cognitive impairment no dementia” | “VR-based cognitive training” | health education | executive function |
| older adults OR elderly OR aged | “virtual reality cognitive rehabilitation” | waitlist | memory |
| predementia |  | physical exercise (non-VR) | visuospatial OR attention |

Study design filter:
“randomized controlled trial” OR RCT

| **List your inclusion criteria – gender, age, year of publication, language** | **List irrelevant terms that you may want to exclude in your search** |
| --- | --- |
| 1. Participants aged ≥60 years with MCI or predementia. 2. Randomized controlled trials. 3. VR-based cognitive training as primary intervention. 4. Non-VR comparator group. 5. Quantitative cognitive outcome measures. 6. Published in English. 7. Published between 1 January 2020 and 31 March 2025. | 1. Diagnosed dementia or severe neurodegenerative disorders. 2. Cognitive impairment secondary to stroke, Parkinson’s disease, traumatic brain injury, or similar conditions. 3. VR used solely for physical rehabilitation without cognitive training. 4. Non-randomized or observational studies. 5. Conference abstracts, editorials, letters, reviews. |

1. **List where you plan to search**

**Main bibliographic databases:**

- - MEDLINE (Via PubMed)
  - Scopus

**Other databases:**

- - ScienceDirect
  - MDPI Journals

**Additional verification search during revision:**

- - Cochrane Library (CENTRAL)

**Other methods:**

- - Backward reference list screening and forward citation tracking using Scopus.

### 9. Search Restrictions

Language: English only
Publication period: 1 January 2020 – 31 March 2025
Unpublished studies: Not sought
Other methods: Manual reference list screening
