# Supplementary material for: Virtual Reality–Based Cognitive Training to Prevent Cognitive Decline in Older Adults With Mild Cognitive Impairment: A Systematic Review of Randomized Controlled Trials
Source: Geriatr Gerontol Int. 2026 Jun 7;26(6):e70586. doi: 10.1111/ggi.70586 (PMC13243133; doi:10.1111/ggi.70586)
Supplement: Supplementary file 2 — Appendix 2: Supplementary Table—Literature search results; PRISMA flow; Data extraction. [file GGI-26-0-s003.docx]

**Supplementary Table – Literature Search Results**

| No | Database | Search Keywords (Simplified Strategy) | Records Identified | Filters Applied | Records After Filters |
| --- | --- | --- | --- | --- | --- |
| 1 | **PubMed (MEDLINE)** | ("Mild Cognitive Impairment"[Mesh] OR "mild cognitive impairment"[Title/Abstract] OR MCI[Title/Abstract] OR predementia[Title/Abstract]) AND ("Virtual Reality"[Mesh] OR "virtual reality"[Title/Abstract] OR VR[Title/Abstract] OR "immersive virtual reality"[Title/Abstract]) AND (randomized controlled trial[Publication Type] OR randomized[Title/Abstract] OR randomised[Title/Abstract]) | 103 | Year: 2020–2025; English; Humans; MEDLINE | 18 |
| 2 | **Scopus** | TITLE-ABS-KEY ("mild cognitive impairment" OR MCI OR predementia) AND TITLE-ABS-KEY ("virtual reality" OR VR OR "immersive virtual reality") AND TITLE-ABS-KEY (randomized OR randomised) | 149 | Year: 2020–2025; Article; Final; Journal; English | 56 |
| 3 | **ScienceDirect** | ("mild cognitive impairment" OR MCI) AND ("virtual reality" OR VR OR "immersive virtual reality") AND (randomized OR randomised OR "randomized controlled trial") | 831 | Year: 2020–2025; Research articles | 246 |
| 4 | **MDPI Journals** | "mild cognitive impairment" AND "virtual reality" | 40 | Year: 2020–2025; Article | 24 |
| 5 | **Cochrane CENTRAL** | ("mild cognitive impairment" OR MCI) AND "virtual reality" | 136 | Trials; Year: 2020–2025 | 106 |
| 6 | **Reference list screening** | Manual search of references from included studies | 2 | - | 2 |

**Note:**

1. In addition to database searches, backward citation tracking of included studies and relevant reviews was performed. Forward citation searches were also conducted using Scopus to identify additional eligible trials.
2. The search was updated on **06 March 2026**
3. All records were exported into reference management software, where duplicate records were identified and removed prior to screening.
4. Title/abstract screening and full-text eligibility assessment were conducted independently by two reviewers. Disagreements were resolved through discussion and consensus.
5. The review process followed the **Preferred Reporting Items for Systematic Reviews and Meta-Analyses (PRISMA 2020)** guidelines.
6. The review protocol was prospectively registered in the **PROSPERO database CRD420261290617**
7. These databases were selected to ensure broad coverage of biomedical, clinical, and interdisciplinary research related to virtual reality and cognitive rehabilitation.

**PRISMA Flow**

**Identification of studies via databases and registers**

452 records identified through

database searching:

PubMed (MEDLINE) = 18

Scopus = 56

ScienceDirect = 246

MDPI Journal = 24

Cochrane CENTRAL = 106

Reference list screening = 2

Records removed *before screening*:

Duplicate records removed (n = 78 )

**Identification**

Records screened

(n = 374)

Records excluded

(n = 354)

Reports sought for retrieval

(n = 20)

Reports not retrieved

(n = 0 )

**Screening**

Reports excluded (n = 12):

Ineligible study design (n = 5)

Ineligible intervention (n = 1)

Participants aged <60 years (n = 4)

Population not mild cognitive impairment (n = 2)

Reports assessed for eligibility

(n = 20)

Studies included in systematic review (n = 8 )

**Included**

**DATA EXTRACTION**

| **No** | **Author (Year, Country)** | **Sample (I/C)** | **Participants** | **Intervention** | **Immersion Level** | **Control** | **Intervention Period** | **Total Sessions** | **Reported Adverse Events** | **Main Outcomes** | **Key Findings** |
| --- | --- | --- | --- | --- | --- | --- | --- | --- | --- | --- | --- |
| 1 | Torpil et al. (2021, Turkey) | 30 / 31 | Older adults with MCI | VR-based cognitive rehabilitation using Microsoft Kinect games | Non-immersive | Conventional cognitive rehabilitation (CR) intervention | 45 min  2x/week  12 weeks | 24 sessions | None reported | The LOTCA-G | Significantly greater improvements in orientation, visual-spatial perception, visuomotor organization, thinking operation, and attention/concentration. |
| 2 | Kang et al. (2021, South Korea) | 23 / 18 | Older adults with subjective cognitive decline or MCI | Fully Immersive Virtual Reality Cognitive Training | Fully immersive | Routine care only (e.g., standard pharmacotherapy). | 20–30 min  2×/week  4 weeks | 8 | Dizziness, nausea, oculomotor disturbance, disorientation | RCFT, SVLT, TMT-A/B, rsfMRI | Significant improvements in visuospatial function and mood, with changes in functional brain connectivity. |
| 3 | Liao et al. (2020, Taiwan) | 18 / 16 | Community-dwelling older adults with MCI | VR-based physical and cognitive training (simulating IADL tasks like cooking and shopping) | Fully Immersive | Combined physical and cognitive training (CPC) | 60 min  3×/week  84 days | 36 | None reported | MoCA, EXIT-25, CVVLT, PFC activation | Significant improvements in global cognition and delayed verbal recall were found only in the VR group |
| 4 | Zheng et al. (2025, China) | 33 / 33 | Older adults with cognitive frailty and ADL limitations | VR-Based Activities of Daily Living (ADL) Rehabilitation Training | Fully immersive | Usual nursing care; maintained regular lifestyle in nursing home. | 45 min  2×/week  12 weeks | 24 | None reported | FIM, BI, IADL, MMSE, GDS-15 | Significant improvements in cognitive function, activities of daily living, and quality-of-life measures. |
| 5 | Chiu et al. (2023, Taiwan) | 30 / 30 | Older adults with cognitive impairment in LTCFs | Virtual Reality Cognitive Training Intervention (VRCTI) | Fully immersive | Routine long-term care without additional training. | 60 min  1×/week  8 weeks | 8 | None reported | CASI, MMSE, CDT-D, QoL | Significant improvements in cognitive function and quality of life, with high completion rates. |
| 6 | Kwan et al. (2024, Hong Kong) | 146 / 147 | Community-dwelling older adults with cognitive frailty | Virtual Reality Motor-Cognitive Training (VRMCT) | Fully immersive | Usual community care; intervention provided after follow-up period. | 60 min (30 min VR)  2×/week  8 weeks | 16 | Vertigo, headache, difficulty focusing, eyestrain | MoCA, SCWT, TMT-B, gait | Improvements in global cognition, gait, and frailty outcomes, with low rates of VR-related discomfort (0.7–3%). |
| 7 | Park et al. (2020a, South Korea) | 10 / 11 | Patients with amnestic MCI | Culture-Based Virtual Reality (VR) Training Program | Non-immersive | Conventional cognitive rehabilitation (e.g., puzzles, cards, maze tasks). | 30 min  5×/week  6 weeks | 30 | Not reported | K-MMSE, SNSB-D, K-CWST | No statistically significant between-group differences observed in cognitive outcomes. |
| 8 | Park et al. (2020b, South Korea) | 18 / 17 | Older adults with MCI | Virtual Reality-Based Cognitive–Motor Rehabilitation (VRCMR) | Fully immersive | Continued normal daily activities throughout the study. | 30 min  2×/week  12 weeks | 24 | Fatigue, dizziness | MoCA, TMT-A/B, DST | Significant improvements in cognitive and motivation-related outcomes, with high adherence reported. |

**REFERANCE**

Chiu, H.-M., Hsu, M.-C., & Ouyang, W.-C. (2023). Effects of incorporating virtual reality training intervention into health care on cognitive function and wellbeing in older adults with cognitive impairment: A randomized controlled trial. *International Journal of Human–Computer Studies*, *170*, 102957. <https://doi.org/10.1016/j.ijhcs.2022.102957>

Kang, J. M., Kim, N., Lee, S. Y., Woo, S. K., Park, G., Yeon, B. K., Park, J. W., Youn, J.-H., Ryu, S.-H., Lee, J.-Y., & Cho, S.-J. (2021). Effect of Cognitive Training in Fully Immersive Virtual Reality on Visuospatial Function and Frontal-Occipital Functional Connectivity in Predementia: Randomized Controlled Trial. *Journal of Medical Internet Research*, *23*(5), e24526. https://doi.org/10.2196/24526

Kwan, R. Y. C., Liu, J., Sin, O. S. K., Fong, K. N. K., Qin, J., Wong, J. C. Y., & Lai, C. (2024). Effects of virtual reality motor-cognitive training for older people with cognitive frailty: Multicentered randomized controlled trial. *Journal of Medical Internet Research*, *26*, e57809. https://doi.org/10.2196/57809

Liao, Y.-Y., Tseng, H.-Y., Lin, Y.-J., Wang, C.-J., & Hsu, W.-C. (2020). Using virtual reality-based training to improve cognitive function, instrumental activities of daily living and neural efficiency in older adults with mild cognitive impairment. *European Journal of Physical and Rehabilitation Medicine*, *56*(1), 47-57. <https://doi.org/10.23736/S1973-9087.19.05899-4>

Page MJ, et al. BMJ 2021;372:n71. doi: 10.1136/bmj.n71.

Park, J.-H., Liao, Y., Kim, D.-R., Song, S., Lim, J. H., Park, H., Lee, Y., & Park, K. W. (2020). Feasibility and tolerability of a culture-based virtual reality (VR) training program in patients with mild cognitive impairment: A randomized controlled pilot study. *International Journal of Environmental Research and Public Health*, *17*(9), 3030. https://doi.org/10.3390/ijerph17093030

Park, J.-S., Jung, Y.-J., & Lee, G. (2020). Virtual reality-based cognitive–motor rehabilitation in older adults with mild cognitive impairment: A randomized controlled study on motivation and cognitive function. *Healthcare*, *8*(3), 335. https://doi.org/10.3390/healthcare8030335

Torpil, B., Sahin, S., Pekçetin, S., & Uyanık, M. (2021). The effectiveness of a virtual reality-based intervention on cognitive functions in older adults with mild cognitive impairment: A single-blind, randomized controlled trial. *Games for Health Journal*, *10*(2), 109-114. https://doi.org/10.1089/g4h.2020.0086

Zheng, L., Li, X., Xu, Y., Yang, Y., Wan, X., Ma, X., Yao, G., & Li, G. (2025). Effects of virtual reality-based activities of daily living rehabilitation training in older adults with cognitive frailty and activities of daily living impairments: A randomized controlled trial. *JAMDA*, *26*, 105397. https://doi.org/10.1016/j.jamda.2024.105397
