# Supplementary material for: Virtual Reality–Based Cognitive Training to Prevent Cognitive Decline in Older Adults With Mild Cognitive Impairment: A Systematic Review of Randomized Controlled Trials
Source: Geriatr Gerontol Int. 2026 Jun 7;26(6):e70586. doi: 10.1111/ggi.70586 (PMC13243133; doi:10.1111/ggi.70586)
Supplement: Supplementary file 3 — Appendix 3: The JBI critical appraisal tool for RCTs Article 1. The JBI critical appraisal tool for RCTs Article 2. The JBI critical appraisal tool for RCTs Article 3. The JBI critical appraisal tool for RCTs Article 4. The JBI critical appraisal tool for RCTs Article 5. The JBI critical appraisal tool for RCTs Article 6. The JBI critical appraisal tool for RCTs Article 7. The JBI critical appraisal tool for RCTs Article 8. [file GGI-26-0-s002.docx]

###

[The JBI Critical Appraisal Tool for RCTs Article 1 3](#_Toc220312627)

[The JBI Critical Appraisal Tool for RCTs Article 2 1](#_Toc220312628)

[The JBI Critical Appraisal Tool for RCTs Article 3 1](#_Toc220312629)

[The JBI Critical Appraisal Tool for RCTs Article 4 1](#_Toc220312630)

[The JBI Critical Appraisal Tool for RCTs Article 5 1](#_Toc220312631)

[The JBI Critical Appraisal Tool for RCTs Article 6 1](#_Toc220312632)

[The JBI Critical Appraisal Tool for RCTs Article 7 1](#_Toc220312633)

[The JBI Critical Appraisal Tool for RCTs Article 8 1](#_Toc220312634)

## The JBI Critical Appraisal Tool for RCTs Article 1

| **Assessor:** | | **Date of Appraisal:** 15 September 2025 | | **Record Number: 1** | | | | |
| --- | --- | --- | --- | --- | --- | --- | --- | --- |
| **Study Author:** Jorge Oliveira, Pedro Gamito, Teresa Souto, Rita Conde, Maria Ferreira, Tatiana Corotnean, Adriano Fernandes, Henrique Silva, dan Teresa Neto | | **Study Title:** Virtual Reality-Based Cognitive Stimulation on People with Mild to Moderate Dementia due to Alzheimer’s Disease: A Pilot Randomized Controlled Trial | | **Study Year:** 2021 | | | | |
|  | |  | |  | | | | |
| **Internal Validity** | | | **Choice - Comments/Justification** | | **Yes** | **No** | **Unclear** | **N/A** |
| **Bias related to selection and allocation** | | | | | | | | |
| **1** | **Was true randomization used for assignment of participants to treatment groups?** | | Participants were randomly assigned to the experimental and control groups using a pilot randomized controlled trial (RCT) design 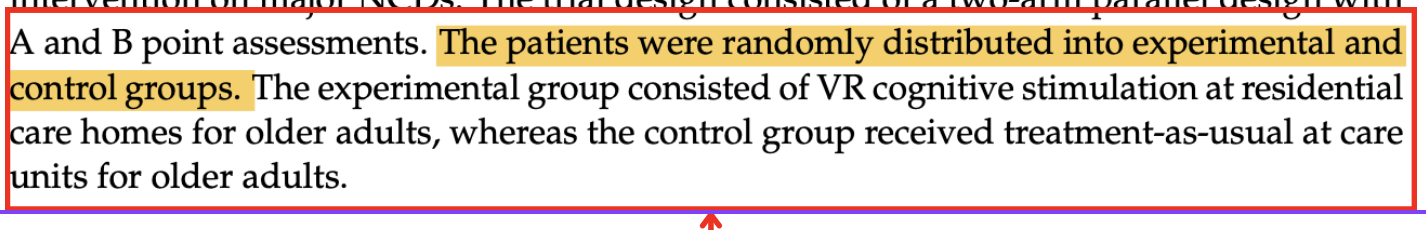 | | ☒ | ☐ | ☐ | ☐ |
| **2** | **Was allocation to treatment groups concealed?** | | The study is described as an **open-label** trial, meaning alocation was likely not concealed from those involved 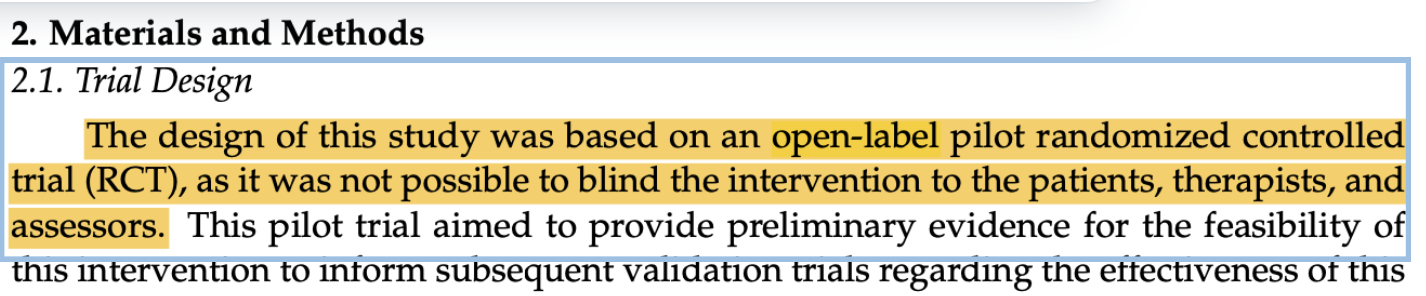 | | ☐ | ☒ | ☐ | ☐ |
| **3** | **Were treatment groups similar at the baseline?** | | There were no statistically significant differences between groups regarding age, gender, education, or baseline clinical dementia ratings (CDR) 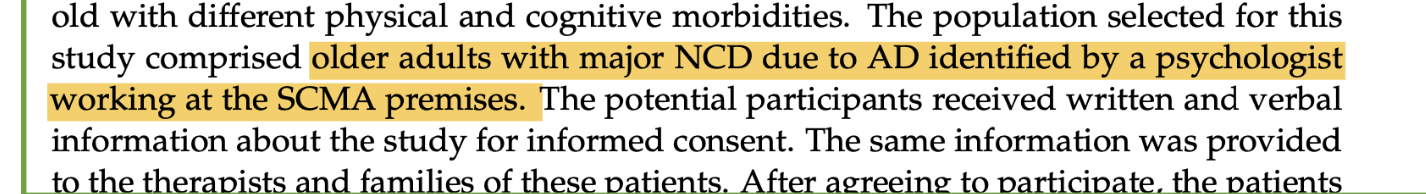 | | ☒ | ☐ | ☐ | ☐ |
| **Bias related to administration of intervention/exposure** | | | | | | | | |
| **4** | **Were participants blind to treatment assignment?** | | Blinding patients to a behavioral/VR intervention was not possible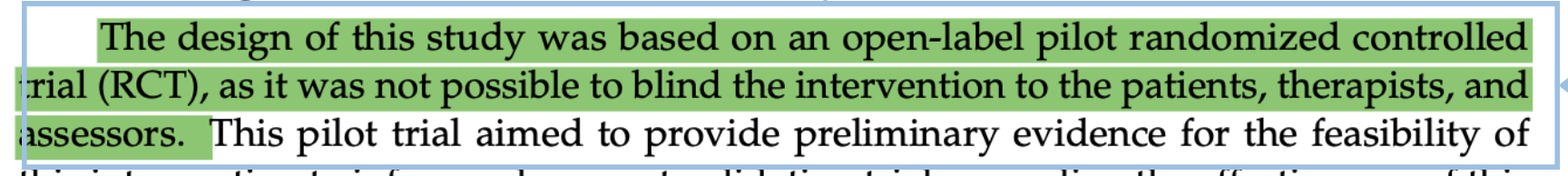 | | ☐ | ☒ | ☐ | ☐ |
| **5** | **Were those delivering the treatment blind to treatment assignment?** | | The therapists delivering the sessions were aware of the group assignments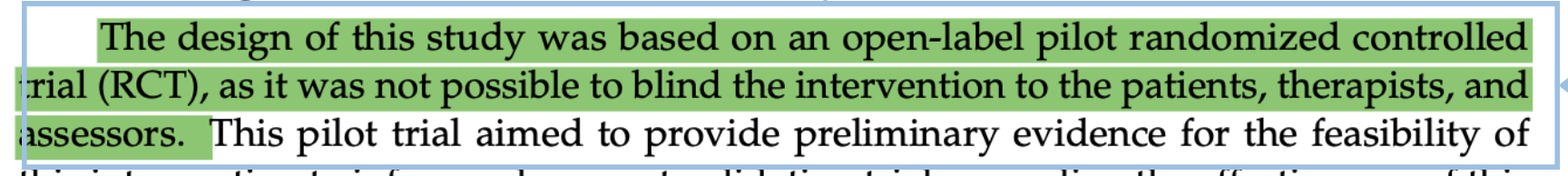 | | ☐ | ☒ | ☐ | ☐ |
| **6** | **Were treatment groups treated identically other than the intervention of interest?** | | The experimental group received VR stimulation sessions (45 min, 2x/week), while the control group received **treatment-as-usual**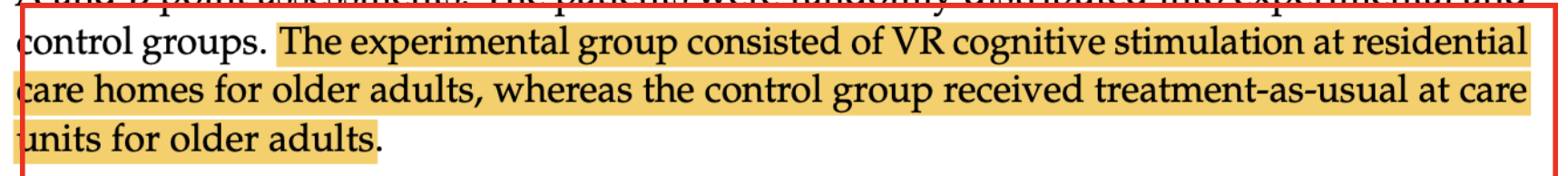 | | ☒ | ☐ | ☐ | ☐ |
| **Bias related to assessment, detection and measurement of the outcome** | | | | | | | | |
| **7** | **Were outcome assessors blind to treatment assignment?** | | 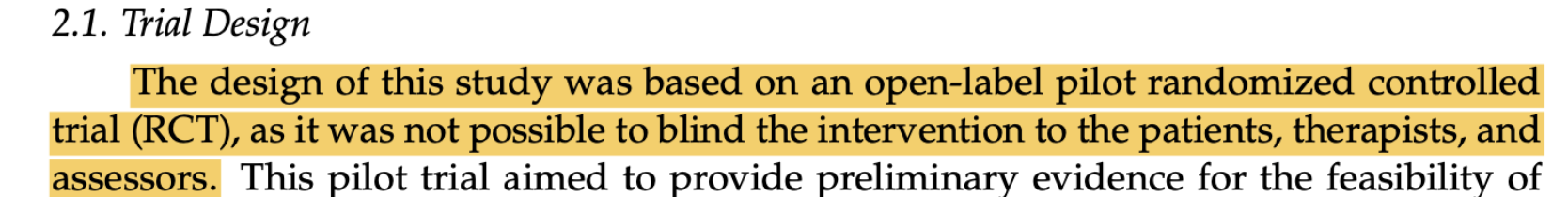 | | **Yes** | **No** | **Unclear** | **N/A** |
|  | **Outcome 1:** Primary Outcomes | | Assessors knew the group assignment during FAB and TMT testing | | ☐ | ☒ | ☐ | ☐ |
|  | **Outcome 2**  Secondary Outcome | | Assessors knew the group assignment during FAB and TMT testing | | ☐ | ☒ | ☐ | ☐ |
|  | **Outcome 3** | |  | | ☐ | ☒ | ☐ | ☐ |
|  | **Outcome 4** | |  | | ☐ | ☒ | ☐ | ☐ |
|  | **Outcome 5** | |  | | ☐ | ☒ | ☐ | ☐ |
|  | **Outcome 6** | |  | | ☐ | ☒ | ☐ | ☐ |
|  | **Outcome 7** | |  | | ☐ | ☒ | ☐ | ☐ |
|  |  | |  | |  |  |  |  |
| **8** | **Were outcomes measured in the same way for treatment groups?** | | 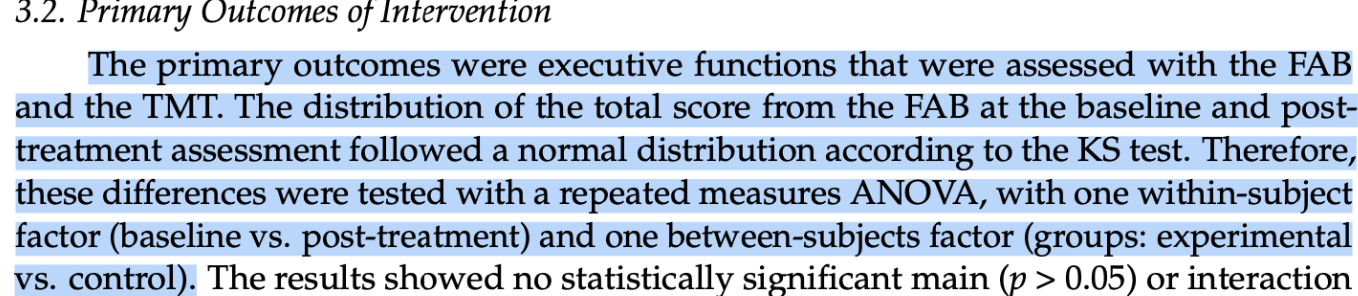 | | **Yes** | **No** | **Unclear** | **N/A** |
|  | **Outcome 1:** Primary Outcomes | | FAB and TMT were applied identically to both groups | | ☒ | ☐ | ☐ | ☐ |
|  | **Outcome 2**  Secondary Outcome | | MMSE and CDT were applied identically to both groups | | ☒ | ☐ | ☐ | ☐ |

| **9** | **Were outcomes measured in a reliable way** | | | | 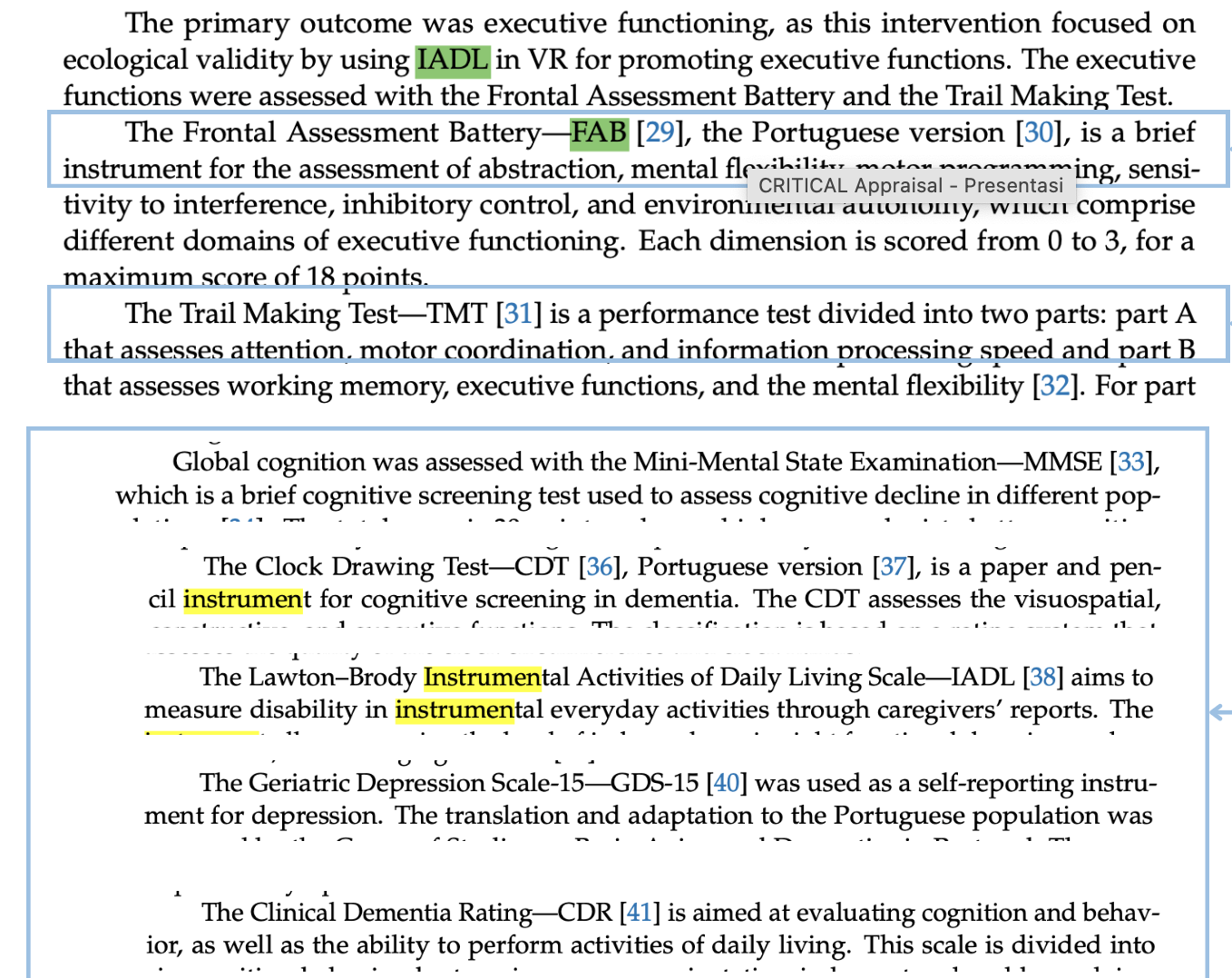 | **Yes** | **No** | | | **Unclear** | | | | **N/A** | | | |
| --- | --- | --- | --- | --- | --- | --- | --- | --- | --- | --- | --- | --- | --- | --- | --- | --- | --- |
|  | **Outcome 1:** Primary Outcomes | | | | The study utilized validated and established measures (MMSE, FAB, TMT, CDT) | ☒ | ☐ | | | ☐ | | | | ☐ | | | |
|  | **Outcome 2**  Secondary Outcome | | | | The study utilized validated and established measures (MMSE, FAB, TMT, CDT) | ☒ | ☐ | | | ☐ | | | | ☐ | | | |
|  | **Outcome 3** | | | |  | ☒ | ☐ | | | ☐ | | | | ☐ | | | |
|  | **Outcome 4** | | | |  | ☒ | ☐ | | | ☐ | | | | ☐ | | | |
|  | **Outcome 5** | | | |  | ☒ | ☐ | | | ☐ | | | | ☐ | | | |
|  | **Outcome 6** | | | |  | ☒ | ☐ | | | ☐ | | | | ☐ | | | |
|  | **Outcome 7** | | | |  | ☒ | ☐ | | | ☐ | | | | ☐ | | | |
|  |  | | | |  |  | | | | | | | | | | | |
| **Bias related to participant retention** | | | | | | | | | | | | | | | | | |
| **10** | **Was follow up complete and if not, were differences between groups in terms of their follow up adequately described and analysed?** | | | | 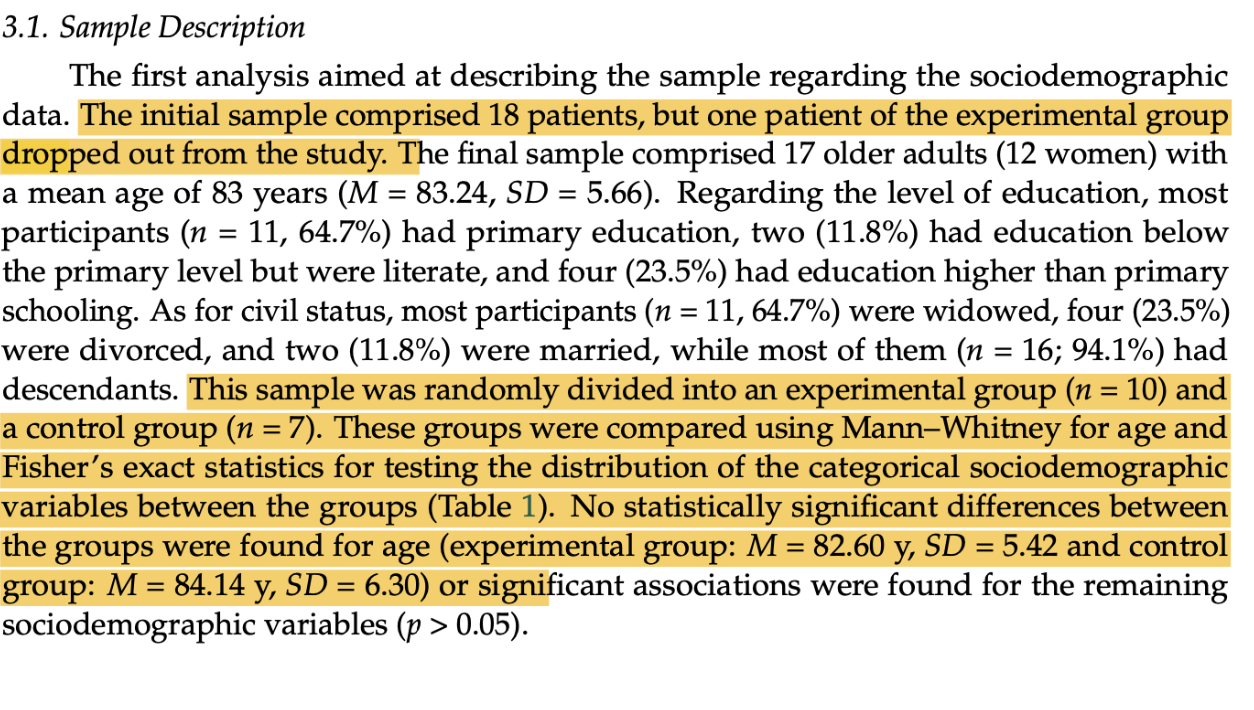 |  | | | | | | | | | | | |
|  | **Outcome 1:** **Primary Outcomes** | | | |  | **Yes** | **No** | | | | **Unclear** | | | **N/A** | | | |
|  |  | Result 1 Executive Function | | | 17/18 participants completed the executive function tests | ☐ | ☐ | | | ☒ | | | | ☐ | | | |
|  | **Outcome 2 Second Outcomes** | | | |  | **Yes** | **No** | | | **Unclear** | | | | **N/A** | | | |
|  |  | Result 1 Global Cognition | | | 17/18 participants completed the global cognition tests | ☐ | ☐ | | | ☒ | | | | ☐ | | | |
|  |  |  |  |  |  |  |  |  |  |  |  |  |  |  |  |  |  |
|  | **Statistical Conclusion Validity** | | | | |  |  | | |  | | | |  | | | |
| **11** | **Were participants analysed in the groups to which they were randomized?** | | | | 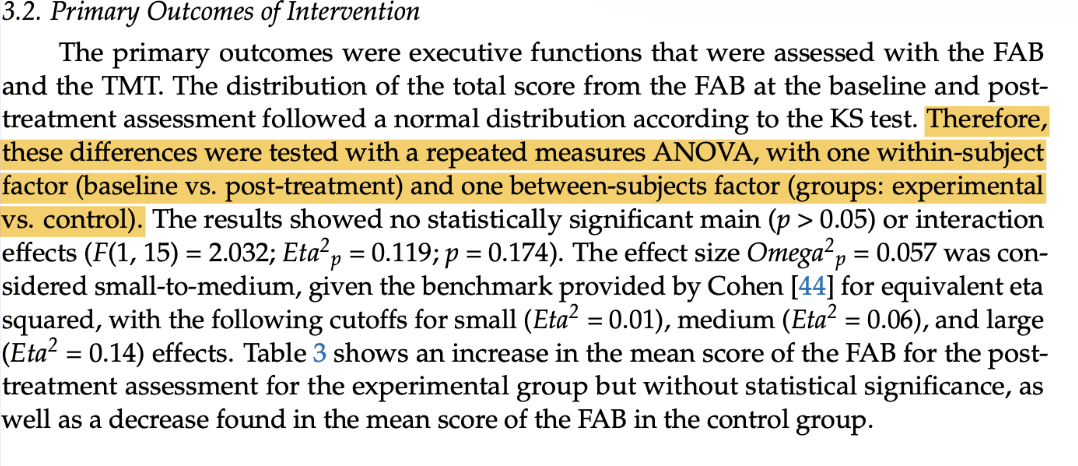 |  | | | | | | | | | | | |
|  | **Outcome 1:** **Primary Outcomes** | | | |  | **Yes** | **No** | | | **Unclear** | | | | **N/A** | | | |
|  |  | Result 1 Executive Function | | | The final analysis included 10 in the experimental group and 7 in the control group as per their initial allocation | ☒ | ☐ | | | ☐ | | | |  | | | |
|  | **Outcome 2 Second Outcomes** | | | |  | **Yes** | **No** | | | **Unclear** | | | | **N/A** | | | |
|  |  | Result 1 Global Cognition | | | The final analysis included 10 in the experimental group and 7 in the control group as per their initial allocation | ☒ | ☐ | | | ☐ | | | |  | | | |
|  |  | | | | |  | |  | | | | |  | |  | | |
| **12** | **Was appropriate statistical analysis used?** | | | | **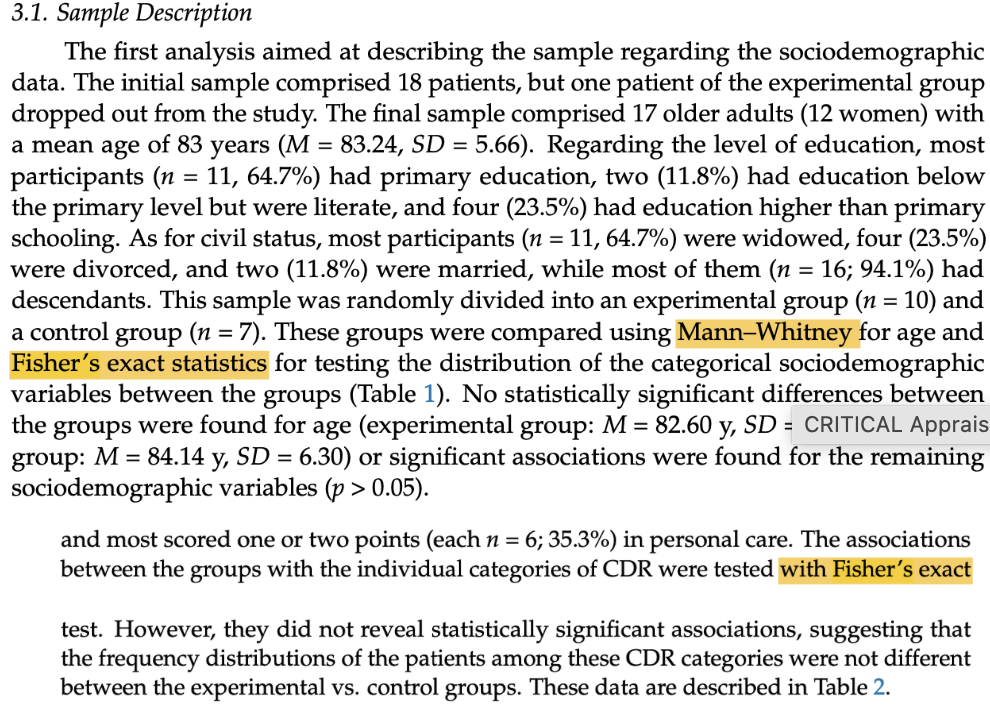** |  | |  | | | | |  | |  | | |
|  | **Outcome 1:** Primary Outcomes | | | | **Outcome 1:** **Primary Outcomes** | **Yes** | | **No** | | | | | **Unclear** | | **N/A** | | |
|  |  | Result 1 Executive Function (FAB) | | | Normality was checked via the Kolmogorov–Smirnov test; repeated measures ANOVA was used for parametric data and Wilcoxon for non-parametric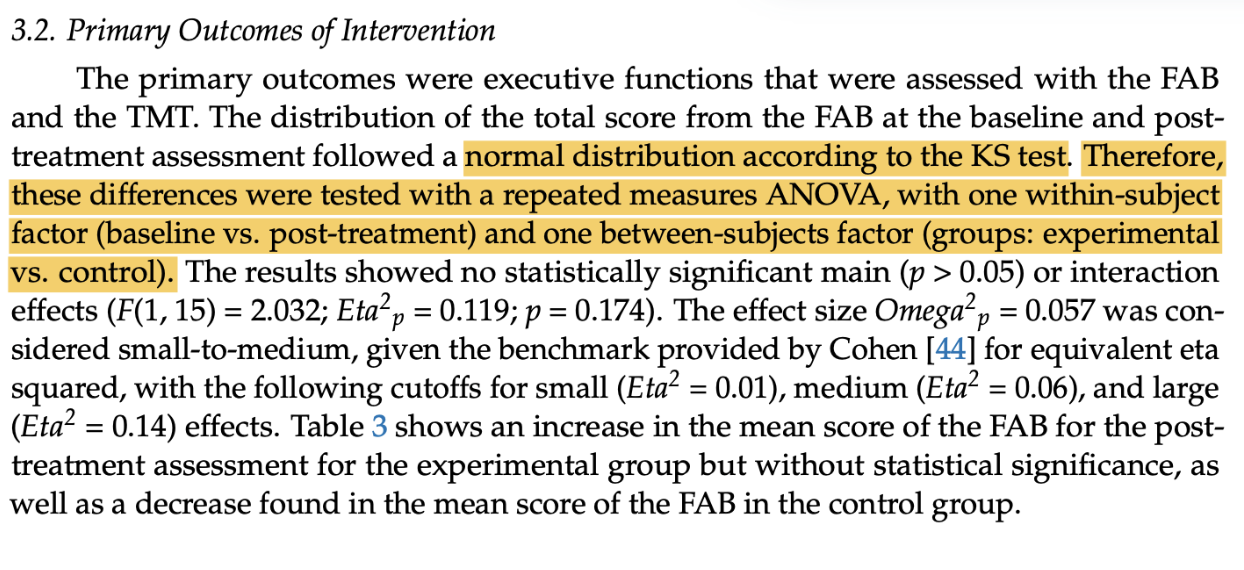 | ☒ | | ☐ | | | | | ☐ | | ☐ | | |
|  |  | Result 2 Executive Function (TMT A & B) | | | Normality was checked via the Kolmogorov–Smirnov test; repeated measures ANOVA was used for parametric data and Wilcoxon for non-parametric | ☒ | | ☐ | | | | ☐ | | | | ☐ | |
|  |  | Result 3 | | |  | ☒ | | ☐ | | | | ☐ | | | | ☐ | |
|  | **Outcome 2** Second Outcomes | | | |  | **Yes** | | **No** | | | | **Unclear** | | | | **N/A** | |
|  |  | Result 1 Global Cognition | | | Normality was checked via the Kolmogorov–Smirnov test; repeated measures ANOVA was used for parametric data and Wilcoxon for non-parametric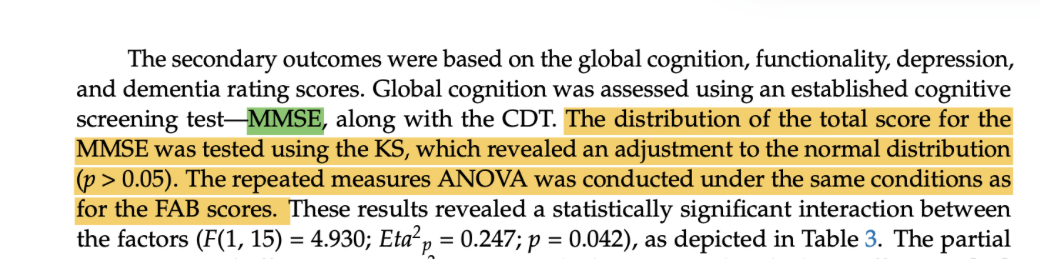 | ☒ | | ☐ | | | | ☐ | | | | ☐ | |
|  |  | | | |  |  | | |  | | |  | | | | |  |
|  |  | | | |  | **Yes** | | | **No** | | | **Unclear** | | | | | **N/A** |
| **13** | **Was the trial design appropriate and any deviations from the standard RCT design (individual randomization, parallel groups) accounted for in the conduct and analysis of the trial?** | | | | It used a two-arm parallel design; however, the lack of an **active control group** was noted as a limitation | ☒ | | | ☐ | | | ☐ | | | | | ☐ |
| **Overall appraisal:** | | | **Include:** ☒ | **Exclude: ☐** | **Seek Further Info: ☐** | | | | | | | | | | | | |
| **Comments:**  The primary limitation regarding internal validity in this study is the **lack of blinding (open-label design)** for participants, therapists, and assessors. This is common in pilot VR studies but increases the risk of performance and detection bias. However, the study maintains strength through **successful randomization** and the use of **validated measurement tools** | | | | | | | | | | | | | | | | | |

## The JBI Critical Appraisal Tool for RCTs Article 2

| **Assessor:** | | | **Date of Appraisal:** 25 September 2025 | | **Record Number: 2** | | | | |
| --- | --- | --- | --- | --- | --- | --- | --- | --- | --- |
| **Study Author:** Jae Myeong Kang, Nambeom Kim, Sook Young Lee, Soo Kyun Woo, Geumjin Park, Byeong Kil Yeon, Jung Woon Park, Jung-Hae Youn, Seung-Ho Ryu, Jun-Young Lee, Seong-Jin Cho | | | **Study Title:** Effect of Cognitive Training in Fully Immersive Virtual Reality on Visuospatial Function and Frontal-Occipital Functional Connectivity in Predementia: Randomized Controlled Trial | | **Study Year:** 2021 | | | | |
|  | | |  | |  | | | | |
| **Internal Validity** | | | | **Choice - Comments/Justification** | | **Yes** | **No** | **Unclear** | **N/A** |
| **Bias related to selection and allocation** | | | | | | | | | |
| **1** | **Was true randomization used for assignment of participants to treatment groups?** | | | Participants were randomly assigned to the VR or control group using a drawing of lots*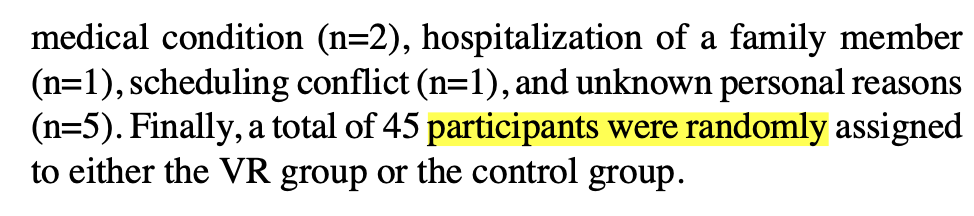* | | ☒ | ☐ | ☐ | ☐ |
| **2** | **Was allocation to treatment groups concealed?** | | | Randomization was performed "with the participants present," which indicates no concealment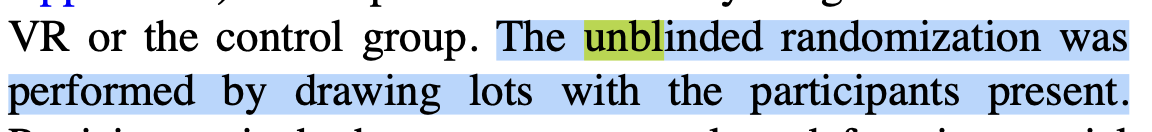 | | ☐ | ☒ | ☐ | ☐ |
| **3** | **Were treatment groups similar at the baseline?** | | | No group differences were found in baseline demographics or diagnostic evaluations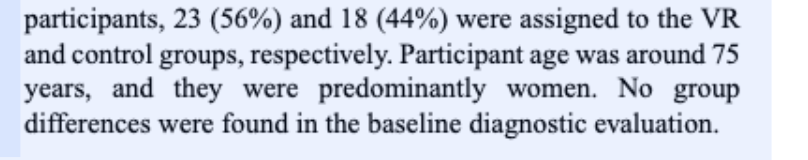 | | ☒ | ☐ | ☐ | ☐ |
| **Bias related to administration of intervention/exposure** | | | | | | | | | |
| **4** | **Were participants blind to treatment assignment?** | | | The study was explicitly described as an **open-label** tria  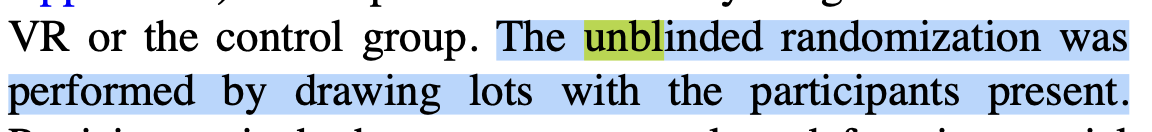 | | ☐ | ☒ | ☐ | ☐ |
| **5** | **Were those delivering the treatment blind to treatment assignment?** | | | Sessions were guided by a certified clinical neuropsychologist, and randomization was unblinded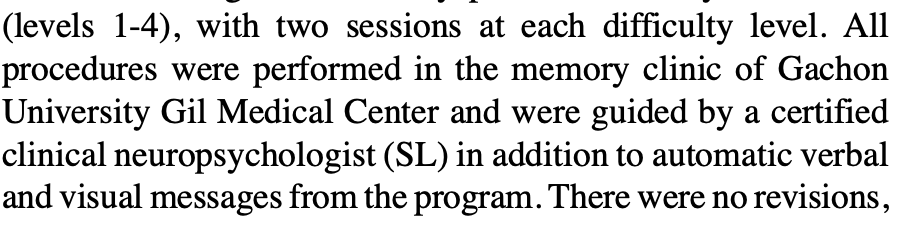 | | ☐ | ☒ | ☐ | ☐ |
| **6** | **Were treatment groups treated identically other than the intervention of interest?** | | | The VR group received additional sessions (2x/week), while the control group only received usual therapy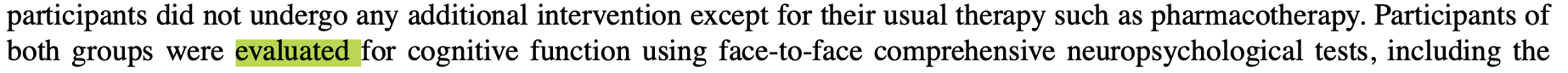 | | ☐ | ☒ | ☐ | ☐ |
| **Bias related to assessment, detection and measurement of the outcome** | | | | | | | | | |
| **7** | **Were outcome assessors blind to treatment assignment?** | | |  | | **Yes** | **No** | **Unclear** | **N/A** |
|  | - Outcome 1 (Visuospatial/RCFT) | | | The neuropsychologist who scored the RCFT copy task was blinded to the randomization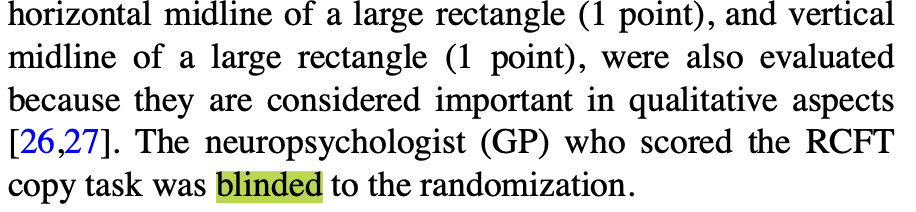 | | ☒ | ☐ | ☐ | ☐ |
|  | - Outcome 2 (Other Psych/Neuro tests) | | | As an open-label trial, general assessments (secondary outcomes) were not stated as blinded | | ☐ | ☒ | ☐ | ☐ |
|  |  | | |  | |  |  |  |  |
| **8** | **Were outcomes measured in the same way for treatment groups?** | | |  | | **Yes** | **No** | **Unclear** | **N/A** |
|  | - Outcome 1 (Visuospatial/RCFT) | | | Measured for both groups at baseline and after 1 month using the RCFT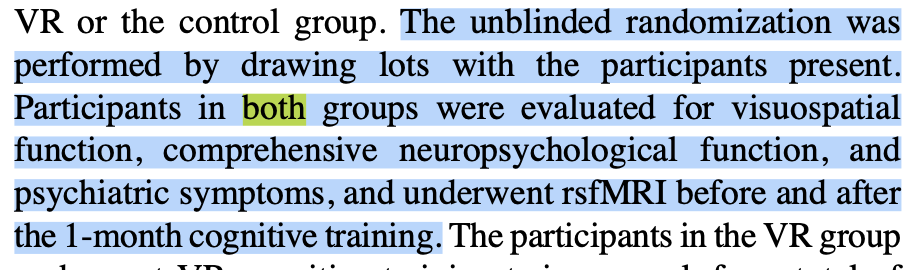 | | ☒ | ☐ | ☐ | ☐ |
|  | - Outcome 2 (Other Psych/Neuro tests) | | | Both groups underwent the same battery of tests (MMSE, GDS, AES, etc.) and rsfMRI | | ☒ | ☐ | ☐ | ☐ |
| ☐ | |  |  |  |  |  |  |  |  |
| ☐ | |  |  |  |  |  |  |  |  |
| ☐ | |  |  |  |  |  |  |  |  |
| ☐ | |  |  |  |  |  |  |  |  |
| ☐ | |  |  |  |  |  |  |  |  |

|  |  | | | |  |  |  | | |  | | | |  | | | |
| --- | --- | --- | --- | --- | --- | --- | --- | --- | --- | --- | --- | --- | --- | --- | --- | --- | --- |
| **9** | **Were outcomes measured in a reliable way** | | | |  | **Yes** | **No** | | | **Unclear** | | | | **N/A** | | | |
|  | - Outcome 1 (Visuospatial/RCFT) | | | | The RCFT copy task is a validated tool in the Korean population | ☒ | ☐ | | | ☐ | | | | ☐ | | | |
|  | - Outcome 2 (Other Psych/Neuro tests) | | | | Used validated and standardized z-scores for all neuropsychological and psychiatric scales | ☒ | ☐ | | | ☐ | | | | ☐ | | | |
|  |  | | | |  |  | | | | | | | | | | | |
| **Bias related to participant retention** | | | | | | | | | | | | | | | | | |
| **10** | **Was follow up complete and if not, were differences between groups in terms of their follow up adequately described and analysed?** | | | |  |  | | | | | | | | | | | |
|  | **Outcome 1:** | | | |  | **Yes** | **No** | | | | **Unclear** | | | **N/A** | | | |
|  |  | - Outcome 1 (Visuospatial/RCFT) | | | 41/45 completed; dropouts were balanced (2 in each group) and reasons were described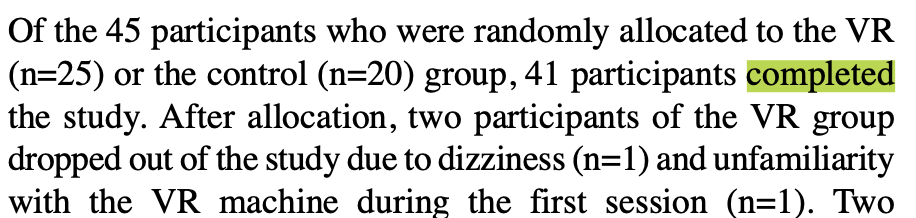  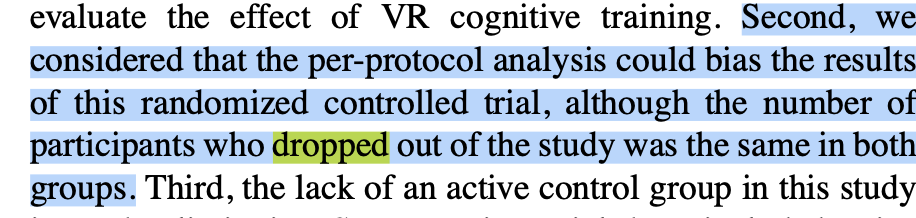 | ☒ | ☐ | | | ☐ | | | | ☐ | | | |
|  |  | Result 2 | | |  | ☐ | ☐ | | | ☐ | | | | ☐ | | | |
|  |  | Result 3 | | |  | ☐ | ☐ | | | ☐ | | | | ☐ | | | |
|  | - Outcome 2 | | | |  | **Yes** | **No** | | | **Unclear** | | | | **N/A** | | | |
|  |  | - Outcome 2 (Other Psych/Neuro tests) | | | All 41 remaining participants completed the full battery of tests and fMRI | ☒ | ☐ | | | ☐ | | | | ☐ | | | |
|  |  |  |  |  |  |  |  |  |  |  |  |  |  |  |  |  |  |
|  | **Statistical Conclusion Validity** | | | | |  |  | | |  | | | |  | | | |
| **11** | **Were participants analysed in the groups to which they were randomized?** | | | |  |  | | | | | | | | | | | |
|  | **Outcome 1:** | | | |  | **Yes** | **No** | | | **Unclear** | | | | **N/A** | | | |
|  |  | - Outcome 1 (Visuospatial/RCFT) | | | The study used **per-protocol analysis**, which the authors noted as a potential bias | ☐ | ☒ | | | ☐ | | | | ☐ | | | |
|  |  | Result 2 | | |  | ☐ | ☐ | | | ☐ | | | | ☐ | | | |
|  |  | Result 3 | | |  | ☐ | ☐ | | | ☐ | | | | ☐ | | | |
|  | **Outcome 2** | | | |  | **Yes** | **No** | | | **Unclear** | | | | **N/A** | | | |
|  |  | - Outcome 2 (Other Psych/Neuro tests) | | | Analyzed based on the 41 participants who completed the study, rather than ITT | ☐ | ☒ | | | ☐ | | | | ☐ | | | |
|  |  | | | | |  | |  | | | | |  | |  | | |
| **12** | **Was appropriate statistical analysis used?** | | | |  |  | |  | | | | |  | |  | | |
|  | **Outcome 1:** | | | |  | **Yes** | | **No** | | | | | **Unclear** | | **N/A** | | |
|  |  | - Outcome 1 (Visuospatial/RCFT) | | | Repeated-measures analysis of variance (ANOVA) digunakan untuk membandingkan perbedaan skor antara kelompok VR dan kontrol, yang sesuai untuk data yang melibatkan dua pengukuran waktu (sebelum dan sesudah pelatihan). *"Repeated-measures analysis of variance was used to compare the effect of cognitive training between groups."*  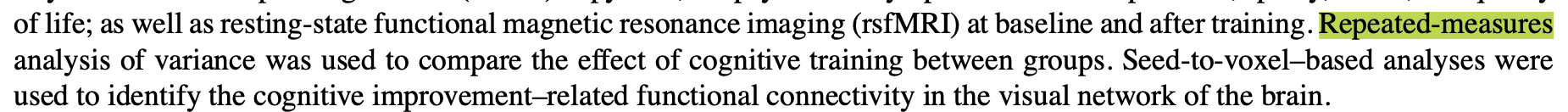 ​ | ☒ | | ☐ | | | | | ☐ | |  | | |
|  |  | Result 2 | | |  | ☐ | | ☐ | | | | ☐ | | | |  | |
|  |  | Result 3 | | |  | ☐ | | ☐ | | | | ☐ | | | |  | |
|  | **Outcome 2** | | | |  | **Yes** | | **No** | | | | **Unclear** | | | | **N/A** | |
|  |  | - Outcome 2 (Other Psych/Neuro tests) | | | Perubahan dalam memori verbal dianalisis dengan cara yang serupa menggunakan ANOVA berulang. *"Repeated-measures analysis of variance was used to compare the effect of cognitive training between groups.* | ☒ | | ☐ | | | | ☐ | | | |  | |
|  |  | | | |  | **Yes** | | | **No** | | | **Unclear** | | | | | **N/A** |
| **13** | **Was the trial design appropriate and any deviations from the standard RCT design (individual randomization, parallel groups) accounted for in the conduct and analysis of the trial?** | | | | Parallel group RCT design was appropriate, and limitations (like lack of active control) were discussed | ☒ | | | ☐ | | | ☐ | | | | | ☐ |
| **Overall appraisal: Yes:** 12  • **No:** 7  • **Unclear:** 0  • **N/A:** 0 | | | **Include:** ☒ | **Exclude: ☐** | **Seek Further Info: ☐** | | | | | | | | | | | | |
| **Comments:**  its main validity limitations stem from the **open-label design** (lack of blinding for participants and therapists) and the use of **per-protocol analysis** instead of Intention-to-Treat,. However, the primary outcome (RCFT) remained strong due to the use of a **blinded assessor** for scoring | | | | | | | | | | | | | | | | | |
|  | | | | | | | | | | | | | | | | | |

## The JBI Critical Appraisal Tool for RCTs Article 3

| **Assessor:** | | | | | **Date of Appraisal:** 30 September 2025 | | **Record Number: 3** | | | | | | | | | | |
| --- | --- | --- | --- | --- | --- | --- | --- | --- | --- | --- | --- | --- | --- | --- | --- | --- | --- |
| **Study Author:** Ying Fu dan Hui Wang | | | | | **Study Title:** Clinical observation of VR virtual reality rehabilitation training combined with acupuncture in the treatment of mild cognitive impairment | | **Study Year:** 2025 | | | | | | | | | | |
|  | | | | |  | |  | | | | | | | | | | |
| **Internal Validity** | | | | | | **Choice - Comments/Justification** | | **Yes** | **No** | | **Unclear** | | | **N/A** | | | |
| **Bias related to selection and allocation** | | | | | | | | | | | | | | | | | |
| **1** | **Was true randomization used for assignment of participants to treatment groups?** | | | | | 48 MCI patients were randomly assigned to the control group and the treatment group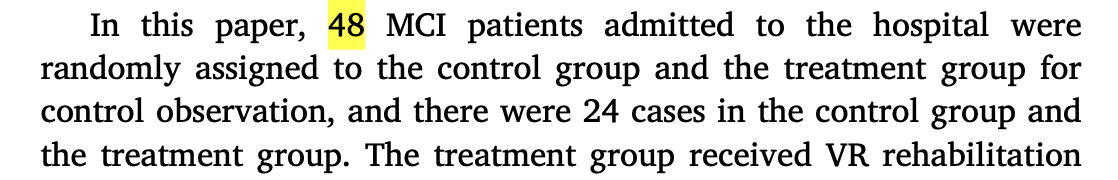 | | ☒ | ☐ | | ☐ | | | ☐ | | | |
| **2** | **Was allocation to treatment groups concealed?** | | | | | The source does not specify if the allocation sequence was concealed from those responsible for recruiting participants. | | ☐ | ☐ | | ☒ | | | ☐ | | | |
| **3** | **Were treatment groups similar at the baseline?** | | | | | There was no significant difference in the basic conditions (gender, age, disease course, education) between the two groups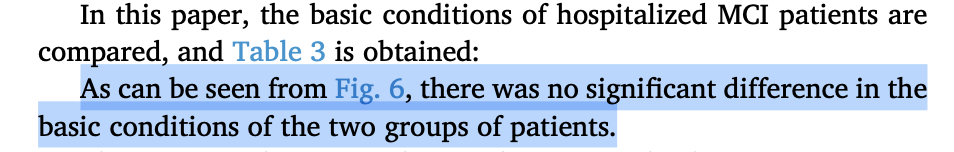 | | ☒ | ☐ | | ☐ | | | ☐ | | | |
| **Bias related to administration of intervention/exposure** | | | | | | | | | | | | | | | | | |
| **4** | **Were participants blind to treatment assignment?** | | | | | As a clinical observation involving physical acupuncture and VR training, blinding participants is generally not feasible and was not reported | | ☐ | ☒ | | ☐ | | | ☐ | | | |
| **5** | **Were those delivering the treatment blind to treatment assignment?** | | | | | The treatment was performed by experienced Chinese medicine practitioners who were aware of the intervention*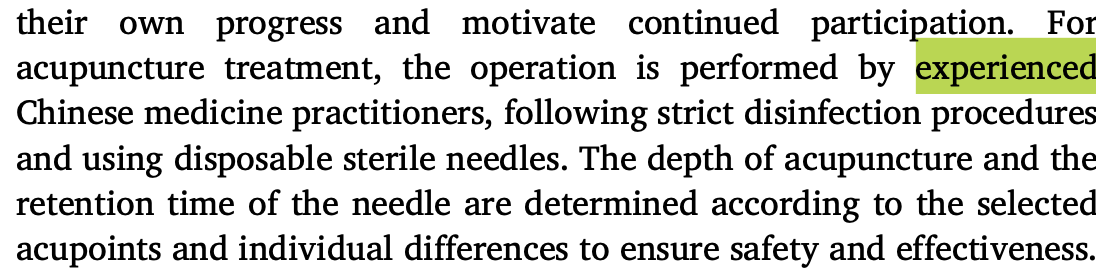*  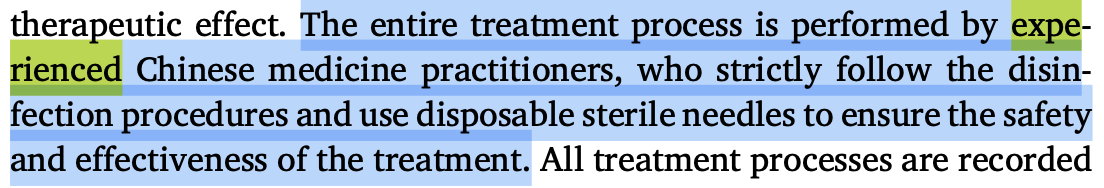 | | ☐ | ☒ | | ☐ | | | ☐ | | | |
| **6** | **Were treatment groups treated identically other than the intervention of interest?** | | | | | The treatment group received VR combined with acupuncture, while the control group received VR training only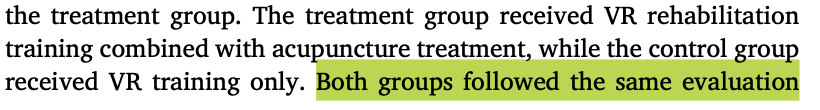  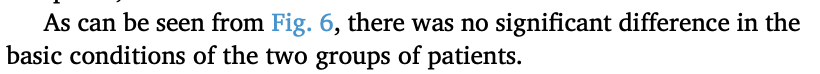 | | ☒ | ☐ | | ☐ | | | ☐ | | | |
| **Bias related to assessment, detection and measurement of the outcome** | | | | | | | | | | | | | | | | | |
| **7** | **Were outcome assessors blind to treatment assignment?** | | | | |  | | **Yes** | **No** | | **Unclear** | | | **N/A** | | | |
|  | **Outcome 1:** MMSE | | | | | It is not stated whether the researchers assessing the MMSE scores were blinded to the group assignment. | | ☐ | ☐ | | ☒ | | | ☐ | | | |
|  | **Outcome 2**  MOCA | | | | | It is not stated whether the researchers assessing the MoCA scores were blinded to the group assignment. | | ☐ | ☐ | | ☒ | | | ☐ | | | |
|  |  | | | | |  | |  |  | |  | | |  | | | |
| **8** | **Were outcomes measured in the same way for treatment groups?** | | | | |  | | **Yes** | **No** | | **Unclear** | | | **N/A** | | | |
|  | **Outcome 1:** MMSE | | | | | Both groups followed the same evaluation criteria and assessment tools for MMSE | | ☒ | ☐ | | ☐ | | | ☐ | | | |
|  | **Outcome 2**  MoCA | | | | | Both groups followed the same evaluation criteria and assessment tools for MoCA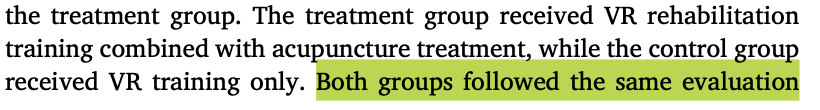  *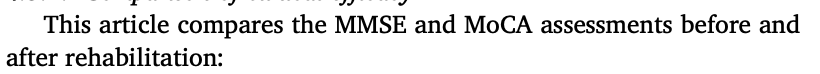*  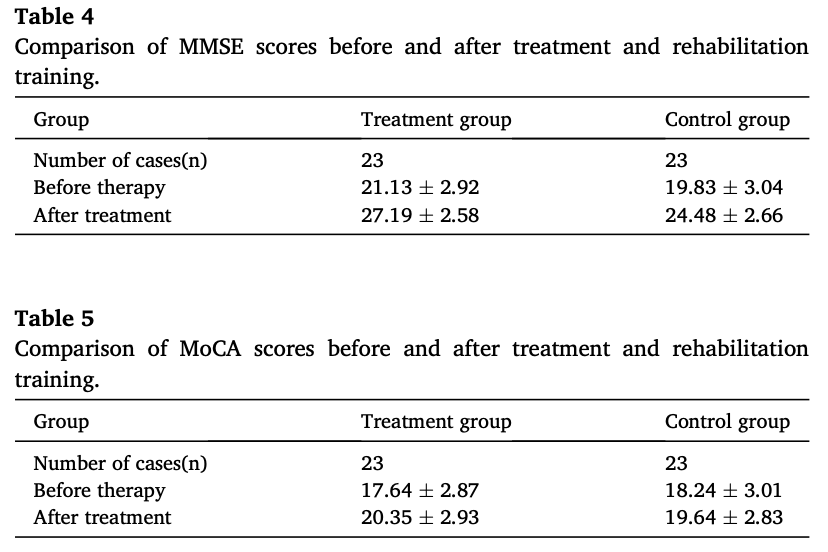 | | ☒ | ☐ | | ☐ | | | ☐ | | | |
|  |  | | | | |  | |  |  | |  | | |  | | | |
| **9** | **Were outcomes measured in a reliable way** | | | | |  | | **Yes** | **No** | | **Unclear** | | | **N/A** | | | |
|  | **Outcome 1:** MMSE | | | | | - MMSE is described as a main scale for measuring MCI in clinical efficacy evaluation | | ☒ | ☐ | | ☐ | | | ☐ | | | |
|  | **Outcome 2**  MoCA | | | | | MoCA is described as a main scale for measuring MCI and was used to evaluate efficacy | | ☒ | ☐ | | ☐ | | | ☐ | | | |
|  |  | | | | |  | |  | | | | | | | | | |
| **Bias related to participant retention** | | | | | | | | | | | | | | | | | |
| **10** | **Was follow up complete and if not, were differences between groups in terms of their follow up adequately described and analysed?** | | | | |  | |  | | | | | | | | | |
|  | **Outcome 1:** | | | | |  | | **Yes** | **No** | | **Unclear** | | | **N/A** | | | |
|  |  | - Outcome 1 (MMSE) | | | | 46 out of 48 cases were effectively completed; dropouts (1 from each group) were explicitly mentioned | | ☒ | ☐ | | ☐ | | | ☐ | | | |
|  |  | Result 2 | | | |  | | ☐ | ☐ | | ☐ | | | ☐ | | | |
|  |  | Result 3 | | | |  | | ☐ | ☐ | | ☐ | | | ☐ | | | |
|  | **Outcome 2** | | | | |  | | **Yes** | **No** | | **Unclear** | | | **N/A** | | | |
|  |  | - Outcome 2 (MoCA) | | | | 46 out of 48 cases were effectively completed; dropouts (1 from each group) were explicitly mentioned | | ☒ | ☐ | | ☐ | | | ☐ | | | |
|  |  | Result 2 | | | |  | | ☐ | ☐ | | ☐ | | | ☐ | | | |
|  | **Statistical Conclusion Validity** | | | | | | |  |  | |  | | |  | | | |
| **11** | **Were participants analysed in the groups to which they were randomized?** | | | | |  | |  | | | | | | | | | |
|  | **Outcome 1:** **Primary Outcomes dan Second Outcomes** | | | | |  | | **Yes** | **No** | | **Unclear** | | | **N/A** | | | |
|  |  | - Outcome 1 (MMSE) | | | | The analysis was conducted on the 46 cases that "effectively completed" the trial, rather than an Intention-To-Treat analysis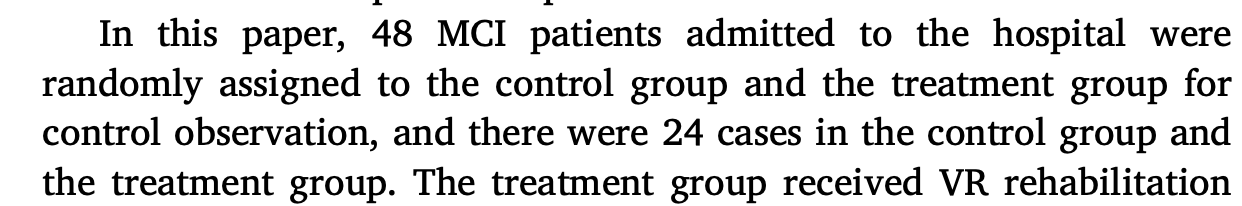 | | ☐ | ☒ | | ☐ | | |  | | | |
|  |  | Result 2 | | | |  | | ☐ | ☐ | | ☐ | | |  | | | |
|  |  | Result 3 | | | |  | | ☐ | ☐ | | ☐ | | |  | | | |
|  | **Outcome 2 Second Outcomes** | | | | |  | | **Yes** | **No** | | **Unclear** | | | **N/A** | | | |
|  |  | - Outcome 2 (MoCA) | | | | The analysis was conducted on the 46 cases that "effectively completed" the trial | | ☐ | ☒ | | ☐ | | |  | | | |
|  |  | Result 2 | | | |  | | ☐ | ☐ | | ☐ | | |  | | | |
|  |  | | | | | | |  |  | | | |  | |  | | |
| **12** | **Was appropriate statistical analysis used?** | | | | |  | |  |  | | | |  | |  | | |
|  | **Outcome 1:** **Primary Outcomes dan Second Outcomes** | | | | |  | | **Yes** | **No** | | | | **Unclear** | | **N/A** | | |
|  |  | - Outcome 1 (MMSE) | | | | - Data analysis was conducted through analysis of variance (ANOVA) and a fixed-effect model for MMSE - "Statistical analysis results. Analysis Indicator Treatment Group Control Group Statistical Method F value P value. MMSE Score Improvement 0.2868 0.105 ANOVA 12.34 < 0.001. MoCA Score Improvement 0.1536 0.062 ANOVA 9.87 < 0.01." - "The total effective rate between the experimental treatment group and the control group was statistically significant (P < 0.0001) ... this paper adopts a random effect model, WMD = 2.42, 95 %CI is (1.98, 3.06). | | ☒ | ☐ | | | | ☐ | | ☐ | | |
|  |  | Result 2 | | | |  | | ☐ | ☐ | | | ☐ | | | | ☐ | |
|  |  | Result 3 | | | |  | | ☐ | ☐ | | | ☐ | | | | ☐ | |
|  | **Outcome 2** | | | | |  | | **Yes** | **No** | | | **Unclear** | | | | **N/A** | |
|  |  | - Outcome 2 (MoCA) | | | | A random-effect model and analysis of covariance (ANCOVA) were used for MoCA and multidimensional cognitive assessments | | ☐ | ☐ | | | ☐ | | | | ☐ | |
|  |  | Result 2 | | | |  | | ☐ | ☐ | | | ☐ | | | | ☐ | |
|  |  | Result 3 | | | |  | | ☐ | ☐ | | | ☐ | | | | ☐ | |
|  |  | | | | |  | |  | |  | |  | | | | |  |
|  |  | | | | |  | | **Yes** | | **No** | | **Unclear** | | | | | **N/A** |
| **13** | **Was the trial design appropriate and any deviations from the standard RCT design (individual randomization, parallel groups) accounted for in the conduct and analysis of the trial?** | | | | | The trial used a randomized parallel-group design with established MCI diagnostic criteria  :  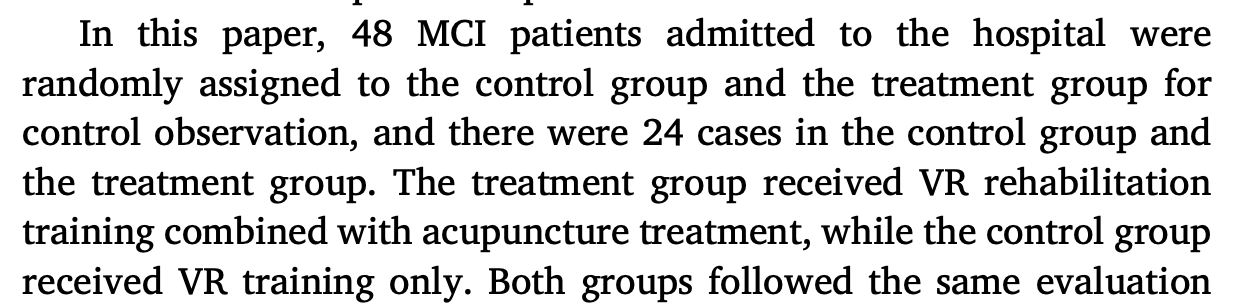 | | ☒ | | ☐ | | ☐ | | | | | ☐ |
| **Overall appraisal:**  • **Yes:** 8  • **No:** 4 (Item 4, 5, 6, and 11)  • **Unclear:** 1  • **N/A:** 0 | | | **Include:** ☒ | **Exclude: ☐** | | **Seek Further Info: ☐** | | | | | | | | | | | |
| **Comments:**  This study demonstrates **strong validity** in terms of **randomization** and **baseline comparability** between groups. However, as is common in many **physical intervention studies**, there is a potential risk of bias due to the **lack of blinding** of participants and practitioners. In addition, the study employed a **per-protocol analysis** (including only participants who completed the intervention) rather than an **Intention-To-Treat (ITT)** approach, which may further increase the risk of bias. | | | | | | | | | | | | | | | | | |

## The JBI Critical Appraisal Tool for RCTs Article 4

| **Assessor:** | | | | | **Date of Appraisal:** 10 November 2025 | | **Record Number: 4** | | | | | | | | | | |
| --- | --- | --- | --- | --- | --- | --- | --- | --- | --- | --- | --- | --- | --- | --- | --- | --- | --- |
| **Study Author:** Lishuang Zheng MSc, Xin Li MSc, Yiran Xu MSc, Yali Yang MSc, Xinyu Wan, Xuehan Ma MSc, Gengxin Yao MSc, Guichen Li PhD | | | | | **Study Title:** Effects of Virtual Reality-Based Activities of Daily Living Rehabilitation Training in Older Adults With Cognitive Frailty and Activities of Daily Living Impairments: A Randomized Controlled Trial | | **Study Year:** 2025 | | | | | | | | | | |
|  | | | | |  | |  | | | | | | | | | | |
| **Internal Validity** | | | | | | **Choice - Comments/Justification** | | **Yes** | **No** | | **Unclear** | | | **N/A** | | | |
| **Bias related to selection and allocation** | | | | | | | | | | | | | | | | | |
| **1** | **Was true randomization used for assignment of participants to treatment groups?** | | | | | 48 MCI patients were randomly assigned to the control group and the treatment group.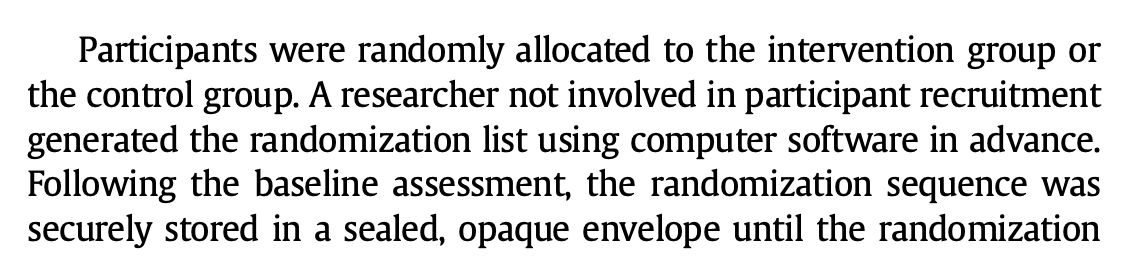 | | ☒ | ☐ | | ☐ | | | ☐ | | | |
| **2** | **Was allocation to treatment groups concealed?** | | | | | "*Following the baseline assessment, the randomization sequence was securely stored in a sealed, opaque envelope until the randomization process was executed*."  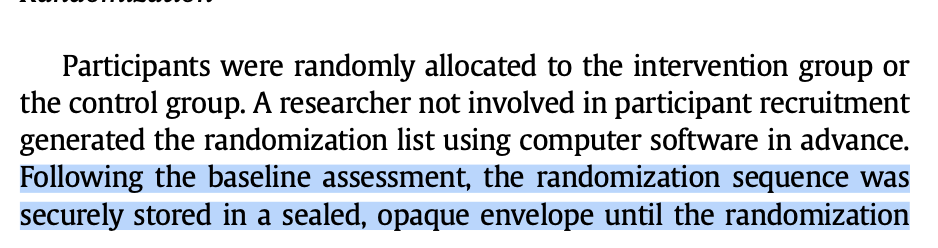 | | ☒ | ☐ | | ☐ | | | ☐ | | | |
| **3** | **Were treatment groups similar at the baseline?** | | | | | No significant difference was found in gender, age, course of disease, or education between groups (p > 0.05).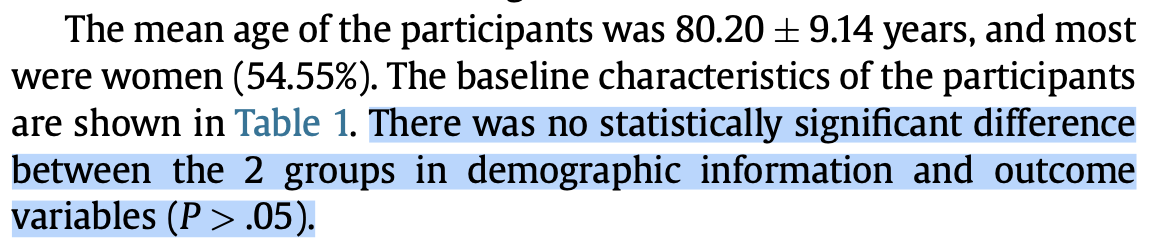 | | ☒ | ☐ | | ☐ | | | ☐ | | | |
| **Bias related to administration of intervention/exposure** | | | | | | | | | | | | | | | | | |
| **4** | **Were participants blind to treatment assignment?** | | | | | The sources explicitly stated that blinding the participants was not feasible | | ☐ | ☒ | | ☐ | | | ☐ | | | |
| **5** | **Were those delivering the treatment blind to treatment assignment?** | | | | | Blinding the interveners (nursing graduate students) was not feasible | | ☐ | ☒ | | ☐ | | | ☐ | | | |
| **6** | **Were treatment groups treated identically other than the intervention of interest?** | | | | | The intervention group received VR training sessions, while the control group received "usual care" and maintained their regular lifestyle | | ☐ | ☒ | | ☐ | | | ☐ | | | |
| **Bias related to assessment, detection and measurement of the outcome** | | | | | | | | | | | | | | | | | |
| **7** | **Were outcome assessors blind to treatment assignment?** | | | | |  | | **Yes** | **No** | | **Unclear** | | | **N/A** | | | |
|  | Outcome 1 (ADL performance: FIM/BI/IADL) | | | | | Outcome data assessors were blinded to the group allocation | | ☒ | ☐ | | ☐ | | | ☐ | | | |
|  | Outcome 2 (Cognitive function: MMSE) | | | | | Data collection was carried out by researchers who were blinded to the group allocation | | ☒ | ☐ | | ☐ | | | ☐ | | | |
|  | **Outcome 3** | | | | |  | | ☐ | ☐ | | ☐ | | | ☐ | | | |
|  | **Outcome 4** | | | | |  | | ☐ | ☐ | | ☐ | | | ☐ | | | |
|  | **Outcome 5** | | | | |  | | ☐ | ☐ | | ☐ | | | ☐ | | | |
|  | **Outcome 6** | | | | |  | | ☐ | ☐ | | ☐ | | | ☐ | | | |
|  | **Outcome 7** | | | | |  | | ☐ | ☐ | | ☐ | | | ☐ | | | |
|  |  | | | | |  | |  |  | |  | | |  | | | |
| **8** | **Were outcomes measured in the same way for treatment groups?** | | | | |  | | **Yes** | **No** | | **Unclear** | | | **N/A** | | | |
|  | - Outcome 1 (ADL performance: FIM/BI/IADL) | | | | | Assessments remained consistent and were collected at three time points (T0, T1, T2) for all groups | | ☒ | ☐ | | ☐ | | | ☐ | | | |
|  | - Outcome 2 (Cognitive function: MMSE) | | | | | Assessments followed the same protocol for both groups at each time point | | ☒ | ☐ | | ☐ | | | ☐ | | | |
|  | **Outcome 3** | | | | |  | | ☐ | ☐ | | ☐ | | | ☐ | | | |
|  | **Outcome 4** | | | | |  | | ☐ | ☐ | | ☐ | | | ☐ | | | |
|  | **Outcome 5** | | | | |  | | ☐ | ☐ | | ☐ | | | ☐ | | | |
|  | **Outcome 6** | | | | |  | | ☐ | ☐ | | ☐ | | | ☐ | | | |
|  | **Outcome 7** | | | | |  | | ☐ | ☐ | | ☐ | | | ☐ | | | |
|  |  | | | | |  | |  |  | |  | | |  | | | |
| **9** | **Were outcomes measured in a reliable way** | | | | |  | | **Yes** | **No** | | **Unclear** | | | **N/A** | | | |
|  | Outcome 1 (ADL performance: FIM/BI/IADL) | | | | | The study used standardized and validated scales (Functional Independence Measure, Barthel Index, and Lawton IADL scale)    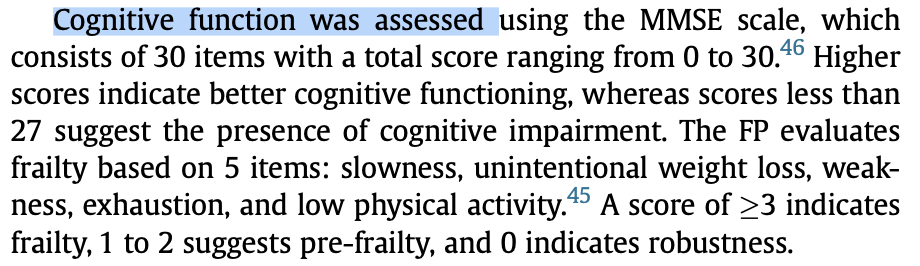 | | ☒ | ☐ | | ☐ | | | ☐ | | | |
|  | - Outcome 2 (Cognitive function: MMSE) | | | | | The Mini-Mental State Examination (MMSE) is a standardized and widely recognized tool for cognitive assessment  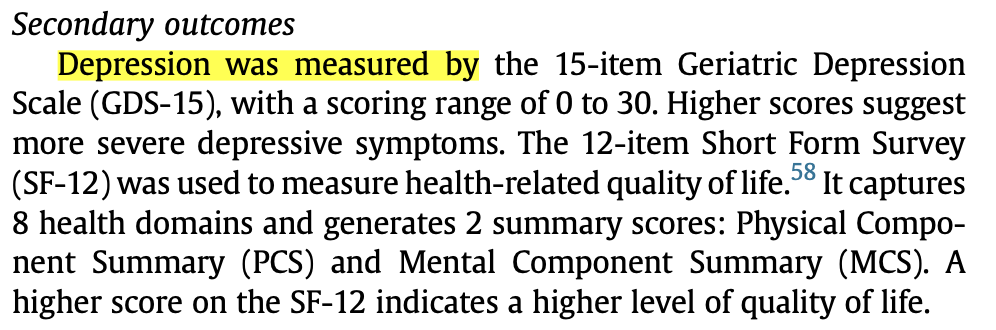 | | ☒ | ☐ | | ☐ | | | ☐ | | | |
|  | **Outcome 3** | | | | |  | | ☐ | ☐ | | ☐ | | | ☐ | | | |
|  | **Outcome 4** | | | | |  | | ☐ | ☐ | | ☐ | | | ☐ | | | |
|  | **Outcome 5** | | | | |  | | ☐ | ☐ | | ☐ | | | ☐ | | | |
|  | **Outcome 6** | | | | |  | | ☐ | ☐ | | ☐ | | | ☐ | | | |
|  | **Outcome 7** | | | | |  | | ☐ | ☐ | | ☐ | | | ☐ | | | |
|  |  | | | | |  | |  | | | | | | | | | |
| **Bias related to participant retention** | | | | | | | | | | | | | | | | | |
| **10** | **Was follow up complete and if not, were differences between groups in terms of their follow up adequately described and analysed?** | | | | |  | |  | | | | | | | | | |
|  | **Outcome 1:** **Primary Outcomes** | | | | |  | | **Yes** | **No** | | **Unclear** | | | **N/A** | | | |
|  |  | Outcome 1 (ADL performance: FIM/BI/IADL) | | | | The attrition rate was 9% (6 participants), and the reasons for dropouts were described. Missing values were imputed using the last observation carried forward (LOCF) method  *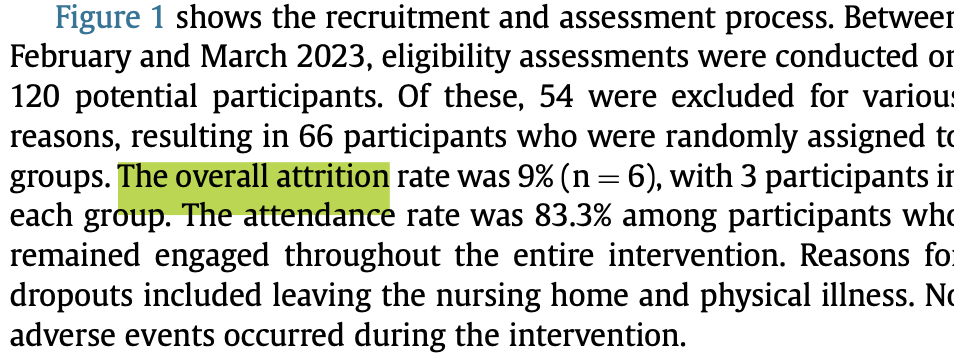* | | ☒ | ☐ | | ☐ | | | ☐ | | | |
|  | **Outcome 2 Secondary Outcomes** | | | | |  | | **Yes** | **No** | | **Unclear** | | | **N/A** | | | |
|  |  | Outcome 2 (Cognitive function: MMSE) | | | | Attrition and missing data were handled using the Intention-To-Treat (ITT) principle for all outcomes | | ☒ | ☐ | | ☐ | | | ☐ | | | |
|  |  |  | | | |  | | ☒ | ☐ | | ☐ | | | ☐ | | | |
|  | **Statistical Conclusion Validity** | | | | | | |  |  | |  | | |  | | | |
| **11** | **Were participants analysed in the groups to which they were randomized?** | | | | |  | |  | | | | | | | | | |
|  | **Outcome 1:** **Primary Outcomes** | | | | |  | | **Yes** | **No** | | **Unclear** | | | **N/A** | | | |
|  |  | Outcome 1 (ADL performance: FIM/BI/IADL) | | | | Analysis followed the Intention-To-Treat (ITT) principle | | ☒ | ☐ | | ☒ | | |  | | | |
|  | **Outcome 2 Second Outcomes** | | | | |  | | **Yes** | **No** | | **Unclear** | | | **N/A** | | | |
|  |  | Outcome 2 (Cognitive function: MMSE) | | | | Analysis followed the Intention-To-Treat (ITT) principle | | ☒ | ☐ | | ☐ | | |  | | | |
|  |  | | | | | | |  |  | | | |  | |  | | |
| **12** | **Was appropriate statistical analysis used?** | | | | |  | |  |  | | | |  | |  | | |
|  | **Outcome 1:** **Primary Outcomes** | | | | |  | | **Yes** | **No** | | | | **Unclear** | | **N/A** | | |
|  |  | Outcome 1 (ADL performance: FIM/BI/IADL) | | | | Generalized Estimating Equations (GEE) were used to examine differential changes | | ☒ | ☐ | | | | ☐ | | ☐ | | |
|  | **Outcome 2 Second Outcomes** | | | | |  | | **Yes** | **No** | | | **Unclear** | | | | **N/A** | |
|  |  | Outcome 2 (Cognitive function: MMSE) | | | | Statistical tests used (GEE, independent t-tests, chi-square) were appropriate for the data types and trial design | | ☒ | ☐ | | | ☐ | | | | ☐ | |
|  |  | | | | |  | |  | |  | |  | | | | |  |
|  |  | | | | |  | | **Yes** | | **No** | | **Unclear** | | | | | **N/A** |
| **13** | **Was the trial design appropriate and any deviations from the standard RCT design (individual randomization, parallel groups) accounted for in the conduct and analysis of the trial?** | | | | | A 2-arm parallel randomized controlled trial (RCT) design was used, following CONSORT guideline | | ☒ | | ☐ | | ☐ | | | | | ☐ |
| **Overall appraisal:**  • **Yes:** 16  • **No:** 3  • **Unclear:** 0  • **N/A:** 0 | | | **Include:** ☒ | **Exclude: ☐** | | **Seek Further Info: ☐** | | | | | | | | | | | |
| **Comments:**  The main strengths of the study by **Zheng et al. (2025)** include the use of an **assessor-blinded double-blind design** and the application of the **Intention-To-Treat (ITT)** principle to manage data from participants who withdrew from the study. Although blinding of participants and intervention providers was not feasible due to the nature of **VR technology**, the study maintained high statistical validity through **computerized randomization** and **allocation concealment using sealed envelopes**. | | | | | | | | | | | | | | | | | |
|  | | | | | | | | | | | | | | | | | |

## The JBI Critical Appraisal Tool for RCTs Article 5

| **Assessor:** | | | | | **Date of Appraisal:** 15 November 2025 | | **Record Number: 5** | | | | | | | | | | |
| --- | --- | --- | --- | --- | --- | --- | --- | --- | --- | --- | --- | --- | --- | --- | --- | --- | --- |
| **Study Author:** Hui-Min Chiu, Mei-Chi Hsu, Wen-Chen Ouyang | | | | | **Study Title:** Effects of incorporating virtual reality training intervention into health care on cognitive function and wellbeing in older adults with cognitive impairment: A randomized controlled trial | | **Study Year:** 2021 | | | | | | | | | | |
|  | | | | |  | |  | | | | | | | | | | |
| **Internal Validity** | | | | | | **Choice - Comments/Justification** | | **Yes** | **No** | | **Unclear** | | | **N/A** | | | |
| **Bias related to selection and allocation** | | | | | | | | | | | | | | | | | |
| **1** | **Was true randomization used for assignment of participants to treatment groups?** | | | | | 60 participants were randomly assigned to either the VR or control groups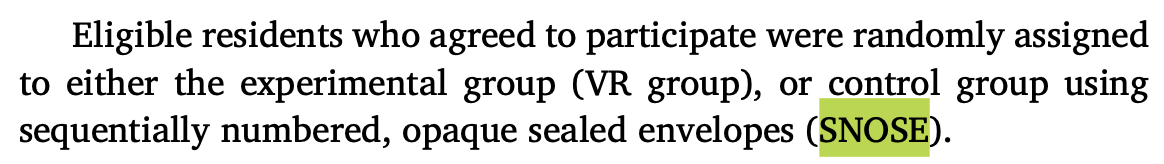 | | ☒ | ☐ | | ☐ | | | ☐ | | | |
| **2** | **Was allocation to treatment groups concealed?** | | | | | Sequentially numbered, opaque sealed envelopes (SNOSE) were used for randomization  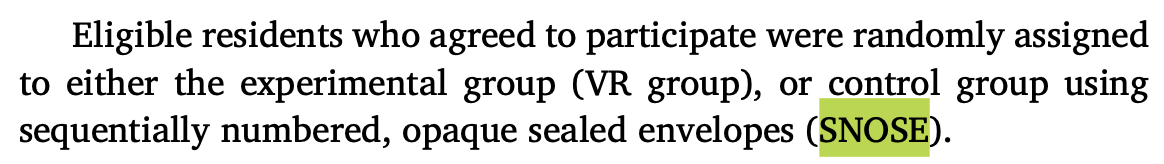 | | ☒ | ☐ | | ☐ | | | ☐ | | | |
| **3** | **Were treatment groups similar at the baseline?** | | | | | Baseline demographics (age, gender, marriage, etc.) and outcome variables showed no significant differences between groups (p > 0.05) | | ☒ | ☐ | | ☐ | | | ☐ | | | |
| **Bias related to administration of intervention/exposure** | | | | | | | | | | | | | | | | | |
| **4** | **Were participants blind to treatment assignment?** | | | | | Participants were aware of the VR intervention; blinding is not feasible for this type of technology | | ☐ | ☒ | | ☐ | | | ☐ | | | |
| **5** | **Were those delivering the treatment blind to treatment assignment?** | | | | | Researchers were present at each session to instruct participants and handle technical functions | | ☐ | ☐ | | ☒ | | | ☐ | | | |
| **6** | **Were treatment groups treated identically other than the intervention of interest?** | | | | | The VR group received 8 weekly sessions, while the control group received "usual care" or engaged in regular activities  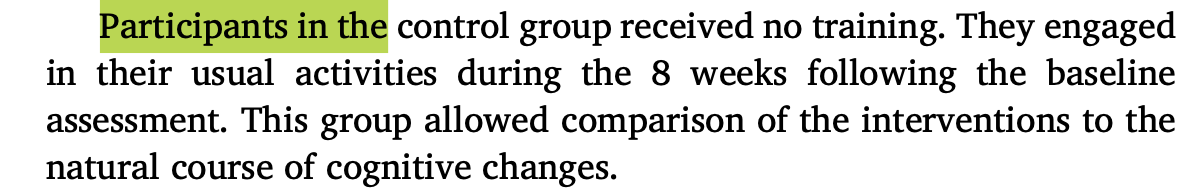 | | ☒ | ☐ | | ☐ | | | ☐ | | | |
| **Bias related to assessment, detection and measurement of the outcome** | | | | | | | | | | | | | | | | | |
| **7** | **Were outcome assessors blind to treatment assignment?** | | | | |  | | **Yes** | **No** | | **Unclear** | | | **N/A** | | | |
|  | - Outcome 1 (Global Cognition: CASI/MMSE) | | | | | The source does not explicitly state that the assessors were blinded to the participants' group assignments.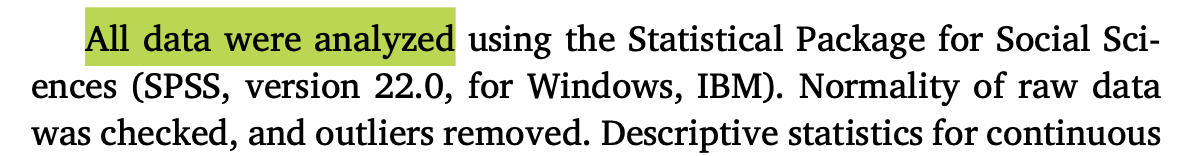 | | ☐ | ☐ | | ☒ | | | ☐ | | | |
|  | - Outcome 2 (Quality of Life: WHOQOL) | | | | | There is no specific mention of blinding for the researchers collecting QOL data. | | ☐ | ☐ | | ☒ | | | ☐ | | | |
|  | **Outcome 3** | | | | |  | | ☐ | ☐ | | ☐ | | | ☐ | | | |
|  | **Outcome 4** | | | | |  | | ☐ | ☐ | | ☐ | | | ☐ | | | |
|  | **Outcome 5** | | | | |  | | ☐ | ☐ | | ☐ | | | ☐ | | | |
|  | **Outcome 6** | | | | |  | | ☐ | ☐ | | ☐ | | | ☐ | | | |
|  | **Outcome 7** | | | | |  | | ☐ | ☐ | | ☐ | | | ☐ | | | |
|  |  | | | | |  | |  |  | |  | | |  | | | |
| **8** | **Were outcomes measured in the same way for treatment groups?** | | | | |  | | **Yes** | **No** | | **Unclear** | | | **N/A** | | | |
|  | - - Outcome 1 (Global Cognition: CASI/MMSE) | | | | | Measures were collected for both groups at baseline and post-test using the same protocols | | ☒ | ☐ | | ☐ | | | ☐ | | | |
|  | - - Outcome 2 (Quality of Life: WHOQOL) | | | | | Measures were collected for both groups at baseline and post-test using the same protocols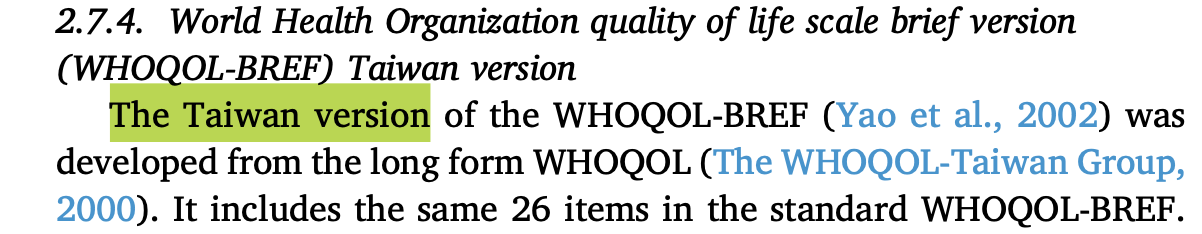 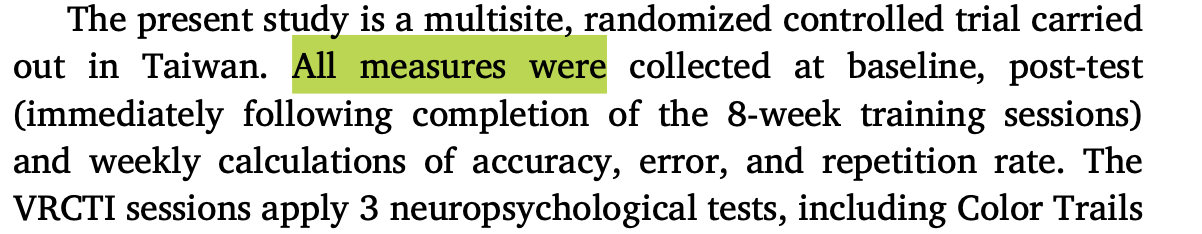  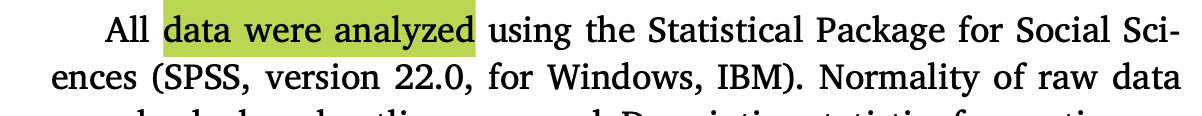 | | ☒ | ☐ | | ☐ | | | ☐ | | | |
|  | **Outcome 3** | | | | |  | | ☐ | ☐ | | ☐ | | | ☐ | | | |
|  | **Outcome 4** | | | | |  | | ☐ | ☐ | | ☐ | | | ☐ | | | |
|  | **Outcome 5** | | | | |  | | ☐ | ☐ | | ☐ | | | ☐ | | | |
|  | **Outcome 6** | | | | |  | | ☐ | ☐ | | ☐ | | | ☐ | | | |
|  | **Outcome 7** | | | | |  | | ☐ | ☐ | | ☐ | | | ☐ | | | |
|  |  | | | | |  | |  |  | |  | | |  | | | |
| **9** | **Were outcomes measured in a reliable way** | | | | |  | | **Yes** | **No** | | **Unclear** | | | **N/A** | | | |
|  | - Outcome 1 (Global Cognition: CASI/MMSE) | | | | | Standardized tools (CASI and Chinese version of MMSE) with established norms were used  *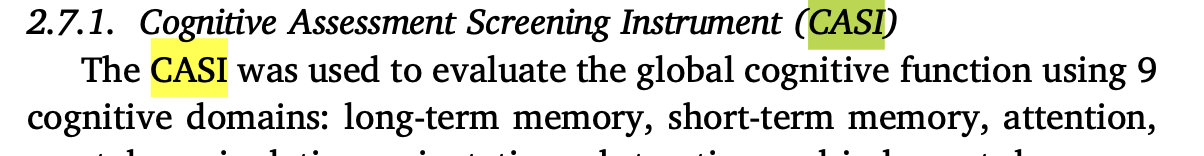*  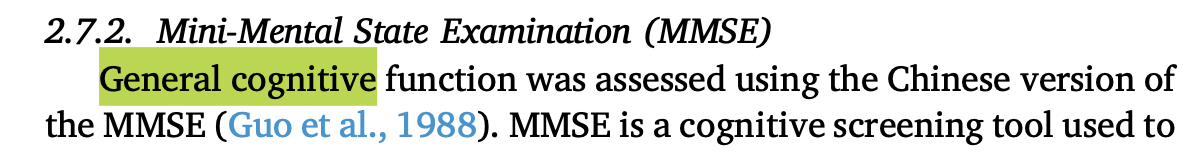  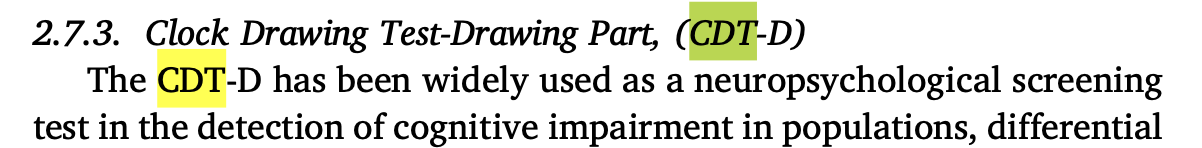 | | ☒ | ☐ | | ☐ | | | ☐ | | | |
|  | - - Outcome 2 (Quality of Life: WHOQOL) | | | | | The validated Taiwan version of the WHOQOL-BREF was used  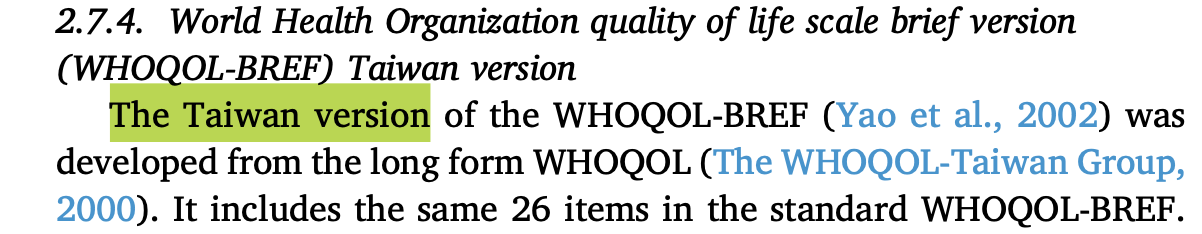  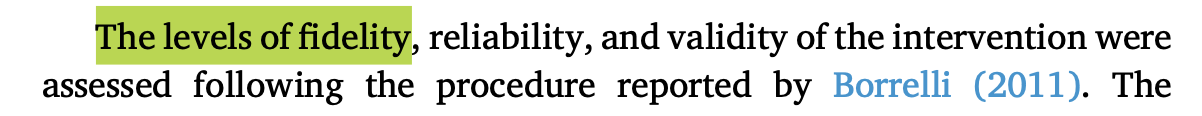 | | ☒ | ☐ | | ☐ | | | ☐ | | | |
|  | **Outcome 3** | | | | |  | | ☐ | ☐ | | ☐ | | | ☐ | | | |
|  | **Outcome 4** | | | | |  | | ☐ | ☐ | | ☐ | | | ☐ | | | |
|  | **Outcome 5** | | | | |  | | ☐ | ☐ | | ☐ | | | ☐ | | | |
|  | **Outcome 6** | | | | |  | | ☐ | ☐ | | ☐ | | | ☐ | | | |
|  | **Outcome 7** | | | | |  | | ☐ | ☐ | | ☐ | | | ☐ | | | |
|  |  | | | | |  | |  | | | | | | | | | |
| **Bias related to participant retention** | | | | | | | | | | | | | | | | | |
| **10** | **Was follow up complete and if not, were differences between groups in terms of their follow up adequately described and analysed?** | | | | |  | |  | | | | | | | | | |
|  | **Outcome 1:** **Primary Outcomes** | | | | |  | | **Yes** | **No** | | **Unclear** | | | **N/A** | | | |
|  |  | - Outcome 1 (Global Cognition: CASI/MMSE) | | | | All 60 participants (30 in VR, 30 in Control) completed the study  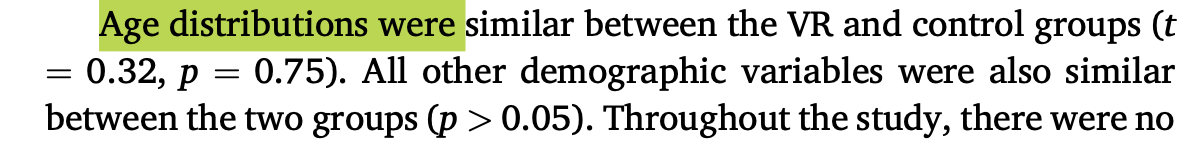 | | ☒ | ☐ | | ☐ | | | ☐ | | | |
|  | **Outcome 2 Secondary Outcomes** | | | | |  | | **Yes** | **No** | | **Unclear** | | | **N/A** | | | |
|  |  | Result 1 Quality of Life - QOL | | | | All 60 participants (30 in VR, 30 in Control) completed the study | | ☒ | ☐ | | ☐ | | | ☐ | | | |
|  |  |  |  |  |  |  |  |  |  |  |  |  |  |  |  |  |  |
|  |  |  |  |  |  |  |  |  |  |  |  |  |  |  |  |  |  |
|  | **Statistical Conclusion Validity** | | | | | | |  |  | |  | | |  | | | |
| **11** | **Were participants analysed in the groups to which they were randomized?** | | | | |  | |  | | | | | | | | | |
|  | **Outcome 1:** **Primary Outcomes** | | | | |  | | **Yes** | **No** | | **Unclear** | | | **N/A** | | | |
|  |  | - Outcome 1 (Global Cognition: CASI/MMSE) | | | | "Eligible residents who agreed to participate were randomly assigned to either the experimental group (VR group), or control group using sequentially numbered, opaque sealed envelopes (SNOSE)"  "Throughout the study, there were no adverse events such as cybersickness, fatigue or dizziness reported (Table 1)" — menunjukkan peserta tetap dalam kelompoknya, tidak ada laporan drop-out karena efek samping  "Post-test CASI scores in the VR group, compared to those in the control group, were significantly improved (p < 0.05) | | ☒ | ☐ | | ☒ | | |  | | | |
|  | **Outcome 2 Second Outcomes** | | | | |  | | **Yes** | **No** | | **Unclear** | | | **N/A** | | | |
|  |  | - Outcome 2 (Quality of Life: WHOQOL) | | | | Quality of Life: "The QOL scores were also significantly higher in the VR group as compared to those in the control group (F = 8.46, p = 0.00, ŋ2 = 0.45)",. | | ☒ | ☐ | | ☐ | | |  | | | |
|  |  | | | | | | |  |  | | | |  | |  | | |
| **12** | **Was appropriate statistical analysis used?** | | | | |  | |  |  | | | |  | |  | | |
|  | **Outcome 1:** **Primary Outcomes** | | | | |  | | **Yes** | **No** | | | | **Unclear** | | **N/A** | | |
|  |  | - Outcome 1 (Global Cognition: CASI/MMSE) | | | | Repeated-measures ANOVA and Generalized Estimating Equations (GEE) were used to analyze changes over time | | ☒ | ☐ | | | | ☐ | | ☐ | | |
|  | **Outcome 2 Second Outcomes** | | | | |  | | **Yes** | **No** | | | **Unclear** | | | | **N/A** | |
|  |  | - Outcome 2 (Quality of Life: WHOQOL) | | | | Repeated-measures ANOVA and Generalized Estimating Equations (GEE) were used to analyze changes over time | | ☒ | ☐ | | | ☐ | | | | ☐ | |
|  |  | | | | |  | |  | |  | |  | | | | |  |
|  |  | | | | |  | | **Yes** | | **No** | | **Unclear** | | | | | **N/A** |
| **13** | **Was the trial design appropriate and any deviations from the standard RCT design (individual randomization, parallel groups) accounted for in the conduct and analysis of the trial?** | | | | | A multisite randomized controlled trial (RCT) design was used | | ☒ | | ☐ | | ☐ | | | | | ☐ |
| **Overall appraisal:**  • **Yes:** 14  • **No:** 3  • **Unclear:** 2  • **N/A:** 0 | | | **Include:** ☒ | **Exclude: ☐** | | **Seek Further Info: ☐** | | | | | | | | | | | |
| **Comments:**  This study possesses high internal validity due to the use of **concealed allocation (SNOSE)**, a **100% participant retention rate** (no dropouts), and the application of robust statistical methods like **GEE**. The primary risks for bias are the lack of blinding for participants and the unclear blinding status of the outcome assessors, which are common limitations in VR clinical trials | | | | | | | | | | | | | | | | | |

## The JBI Critical Appraisal Tool for RCTs Article 6

| **Assessor:** | | | | | **Date of Appraisal:** 20 November 2025 | | **Record Number: 6** | | | | | | | | | | |
| --- | --- | --- | --- | --- | --- | --- | --- | --- | --- | --- | --- | --- | --- | --- | --- | --- | --- |
| **Study Author:** Rick Yiu Cho Kwan, Justina Liu, Olive Suk Kan Sin, Kenneth N K Fong, Jing Qin, Joe Chi Yin Wong, Claudia Lai | | | | | **Study Title:** Effects of Virtual Reality Motor-Cognitive Training for Older People With Cognitive Frailty: Multicentered Randomized Controlled Trial | | **Study Year:** 2024 | | | | | | | | | | |
|  | | | | |  | |  | | | | | | | | | | |
| **Internal Validity** | | | | | | **Choice - Comments/Justification** | | **Yes** | **No** | | **Unclear** | | | **N/A** | | | |
| **Bias related to selection and allocation** | | | | | | | | | | | | | | | | | |
| **1** | **Was true randomization used for assignment of participants to treatment groups?** | | | | | A permuted block randomization with a block size of 8 was generated using computer software.  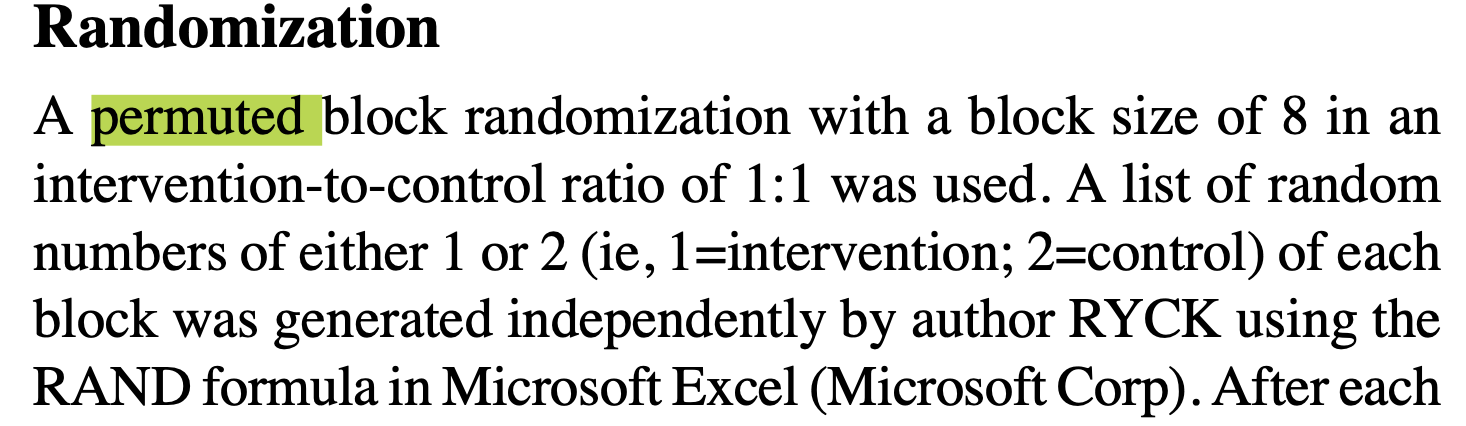 | | ☒ | ☐ | | ☐ | | | ☐ | | | |
| **2** | **Was allocation to treatment groups concealed?** | | | | | Allocation was managed by a research assistant who did not participate in recruitment to ensure concealment. | | ☒ | ☐ | | ☐ | | | ☐ | | | |
| **3** | **Were treatment groups similar at the baseline?** | | | | | Baseline demographics and outcomes were collected for 293 participants; Table 2 shows comparable characteristics between groups.  *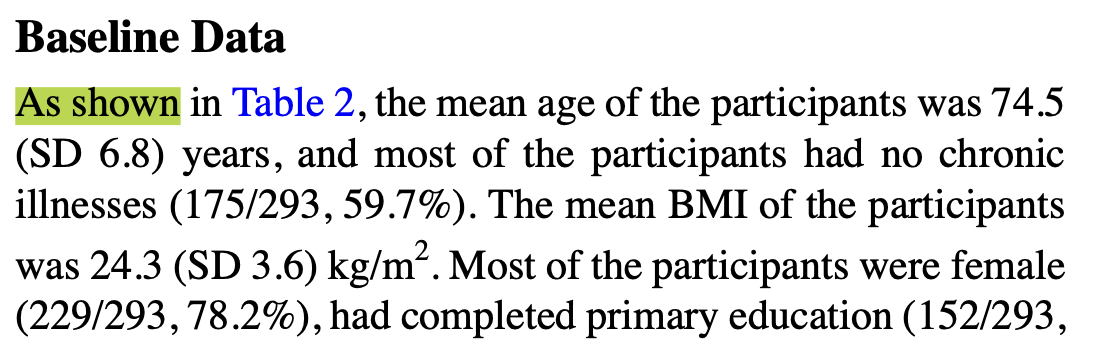* | | ☒ | ☐ | | ☐ | | | ☐ | | | |
| **Bias related to administration of intervention/exposure** | | | | | | | | | | | | | | | | | |
| **4** | **Were participants blind to treatment assignment?** | | | | | It was explicitly stated that it was not possible to blind participants due to the nature of the VR study.*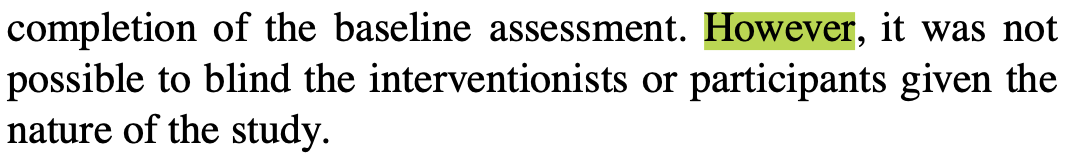* | | ☐ | ☒ | | ☐ | | | ☐ | | | |
| **5** | **Were those delivering the treatment blind to treatment assignment?** | | | | | It was not possible to blind the interventionists (nursing students) delivering the VR training  *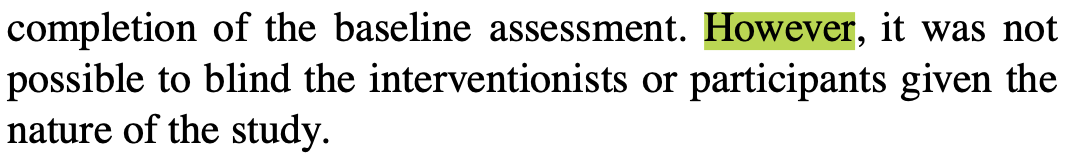* | | ☐ | ☒ | | ☐ | | | ☐ | | | |
| **6** | **Were treatment groups treated identically other than the intervention of interest?** | | | | | The intervention group received VRMCT sessions, while the control group received "usual care" with no interference from investigators    *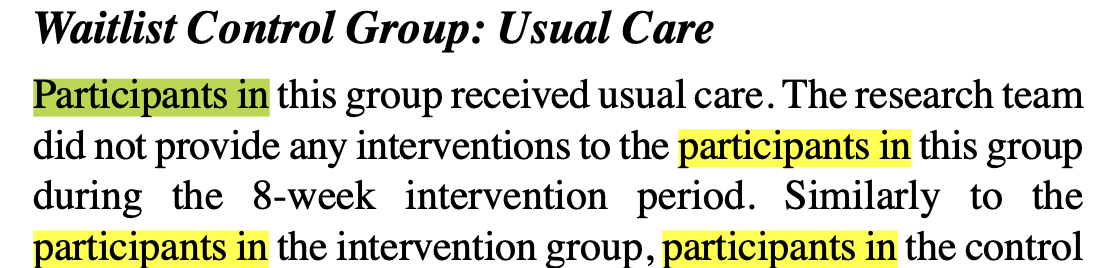* | | ☐ | ☒ | | ☐ | | | ☐ | | | |
| **Bias related to assessment, detection and measurement of the outcome** | | | | | | | | | | | | | | | | | |
| **7** | **Were outcome assessors blind to treatment assignment?** | | | | |  | | **Yes** | **No** | | **Unclear** | | | **N/A** | | | |
|  | - Outcome 1 (Global Cognition: MoCA) | | | | | The study used an assessor-blinded design; all assessors were blinded to group labels  *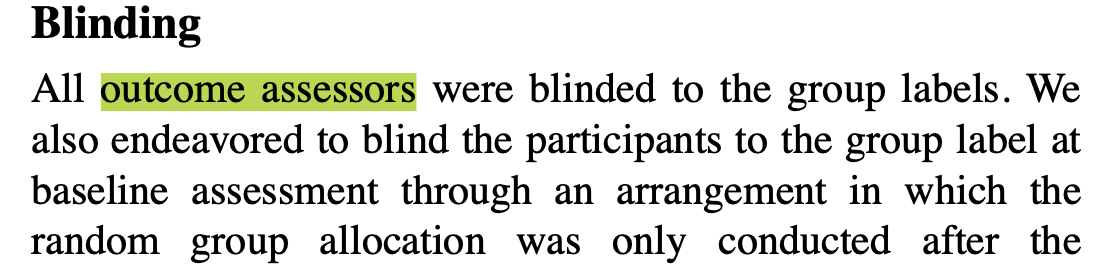* | | ☒ | ☐ | | ☐ | | | ☐ | | | |
|  | - Outcome 2 (Physical Frailty: FFP) | | | | | Assessors were blinded to the group assignments for all outcome measurements | | ☒ | ☐ | | ☐ | | | ☐ | | | |
|  | **Outcome 3** | | | | |  | | ☐ | ☐ | | ☐ | | | ☐ | | | |
|  | **Outcome 4** | | | | |  | | ☐ | ☐ | | ☐ | | | ☐ | | | |
|  | **Outcome 5** | | | | |  | | ☐ | ☐ | | ☐ | | | ☐ | | | |
|  | **Outcome 6** | | | | |  | | ☐ | ☐ | | ☐ | | | ☐ | | | |
|  | **Outcome 7** | | | | |  | | ☐ | ☐ | | ☐ | | | ☐ | | | |
|  |  | | | | |  | |  |  | |  | | |  | | | |
| **8** | **Were outcomes measured in the same way for treatment groups?** | | | | |  | | **Yes** | **No** | | **Unclear** | | | **N/A** | | | |
|  | - - Outcome 1 (Global Cognition: MoCA) | | | | | MoCA was collected at baseline (T0) and post-intervention (T1) using the same protocol for both groups. | | ☒ | ☐ | | ☐ | | | ☐ | | | |
|  | - - Outcome 2 (Physical Frailty: FFP) | | | | | FFP was collected at baseline (T0) and post-intervention (T1) using the same protocol for both groups. | | ☒ | ☐ | | ☐ | | | ☐ | | | |
|  | **Outcome 3** | | | | |  | | ☐ | ☐ | | ☐ | | | ☐ | | | |
|  | **Outcome 4** | | | | |  | | ☐ | ☐ | | ☐ | | | ☐ | | | |
|  | **Outcome 5** | | | | |  | | ☐ | ☐ | | ☐ | | | ☐ | | | |
|  | **Outcome 6** | | | | |  | | ☐ | ☐ | | ☐ | | | ☐ | | | |
|  | **Outcome 7** | | | | |  | | ☐ | ☐ | | ☐ | | | ☐ | | | |
|  |  | | | | |  | |  |  | |  | | |  | | | |
| **9** | **Were outcomes measured in a reliable way** | | | | |  | | **Yes** | **No** | | **Unclear** | | | **N/A** | | | |
|  | - Outcome 1 (Global Cognition: MoCA) | | | | | MoCA has good criterion validity in detecting MCI and strong correlation with established tests. | | ☒ | ☐ | | ☐ | | | ☐ | | | |
|  | - - Outcome 2 (Physical Frailty: FFP) | | | | | FFP is a standard, validated clinical measure for assessing physical frailty. | | ☒ | ☐ | | ☐ | | | ☐ | | | |
|  | **Outcome 3** | | | | |  | | ☐ | ☐ | | ☐ | | | ☐ | | | |
|  | **Outcome 4** | | | | |  | | ☐ | ☐ | | ☐ | | | ☐ | | | |
|  | **Outcome 5** | | | | |  | | ☐ | ☐ | | ☐ | | | ☐ | | | |
|  | **Outcome 6** | | | | |  | | ☐ | ☐ | | ☐ | | | ☐ | | | |
|  | **Outcome 7** | | | | |  | | ☐ | ☐ | | ☐ | | | ☐ | | | |
|  |  | | | | |  | |  | | | | | | | | | |
| **Bias related to participant retention** | | | | | | | | | | | | | | | | | |
| **10** | **Was follow up complete and if not, were differences between groups in terms of their follow up adequately described and analysed?** | | | | |  | |  | | | | | | | | | |
|  | **Outcome 1:** **Primary Outcomes** | | | | |  | | **Yes** | **No** | | **Unclear** | | | **N/A** | | | |
|  |  | Outcome 1 (Global Cognition: MoCA) | | | | Loss to follow-up (14.4% in VR, 7.5% in control) was adequately described and attributed to COVID-19 center closures  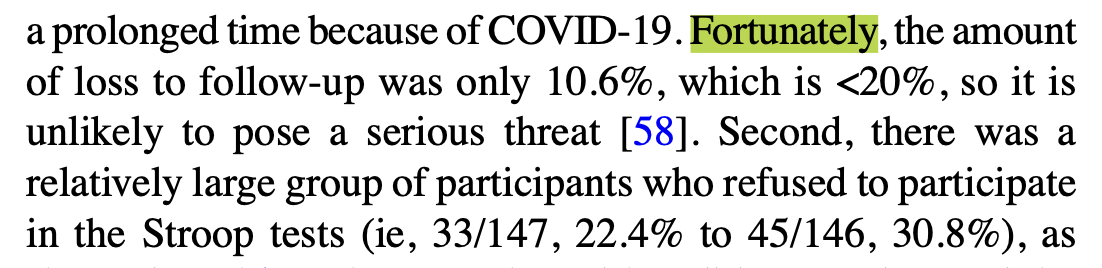  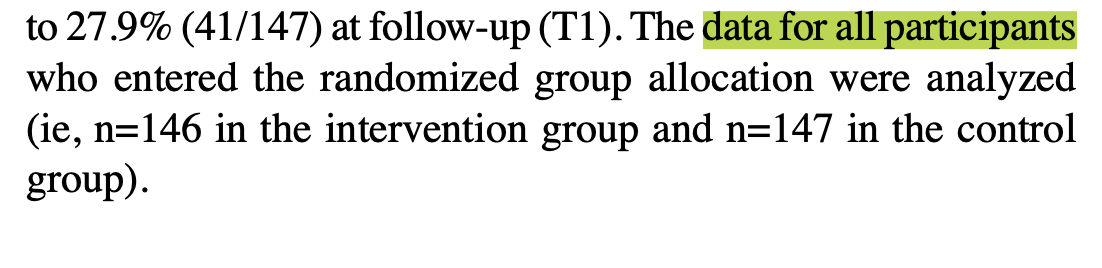 | | ☒ | ☐ | | ☐ | | | ☐ | | | |
|  | **Outcome 2 Secondary Outcomes** | | | | |  | | **Yes** | **No** | | **Unclear** | | | **N/A** | | | |
|  |  | - Outcome 2 (Physical Frailty: FFP) | | | | Missing data were handled using Generalized Estimating Equations (GEE), which estimates missing values | | ☒ | ☐ | | ☐ | | | ☐ | | | |
|  |  |  |  |  |  |  |  |  |  |  |  |  |  |  |  |  |  |
|  | **Statistical Conclusion Validity** | | | | | | |  |  | |  | | |  | | | |
| **11** | **Were participants analysed in the groups to which they were randomized?** | | | | |  | |  | | | | | | | | | |
|  | **Outcome 1:** **Primary Outcomes** | | | | |  | | **Yes** | **No** | | **Unclear** | | | **N/A** | | | |
|  |  | Outcome 1 (Global Cognition: MoCA) | | | | Intention-to-treat (ITT) analysis was adopted; all 293 randomized participants were included in the analysis. | | ☒ | ☐ | | ☒ | | |  | | | |
|  | **Outcome 2 Second Outcomes** | | | | |  | | **Yes** | **No** | | **Unclear** | | | **N/A** | | | |
|  |  | - Outcome 2 (Physical Frailty: FFP) | | | | All randomized participants were analyzed regardless of their adherence or follow-up status | | ☒ | ☐ | | ☐ | | |  | | | |
|  |  | | | | | | |  |  | | | |  | |  | | |
| **12** | **Was appropriate statistical analysis used?** | | | | |  | |  |  | | | |  | |  | | |
|  | **Outcome 1:** **Primary Outcomes** | | | | |  | | **Yes** | **No** | | | | **Unclear** | | **N/A** | | |
|  |  | - Outcome 1 (Global Cognition: MoCA) | | | | Generalized estimating equations (GEE) were used to examine group, time, and interaction effects. | | ☒ | ☐ | | | | ☐ | | ☐ | | |
|  | **Outcome 2 Second Outcomes** | | | | |  | | **Yes** | **No** | | | **Unclear** | | | | **N/A** | |
|  |  | - Outcome 2 (Physical Frailty: FFP) | | | | GEE was used to account for repeated measures and group interactions. | | ☒ | ☐ | | | ☐ | | | | ☐ | |
|  |  | | | | |  | |  | |  | |  | | | | |  |
|  |  | | | | |  | | **Yes** | | **No** | | **Unclear** | | | | | **N/A** |
| **13** | **Was the trial design appropriate and any deviations from the standard RCT design (individual randomization, parallel groups) accounted for in the conduct and analysis of the trial?** | | | | | The study followed CONSORT 2010 guidelines for a multicentered, 2-parallel-group randomized controlled trial. | | ☒ | | ☐ | | ☐ | | | | | ☐ |
| **Overall appraisal:**  • **Yes:** 16  • **No:** 3  • **Unclear:** 0  • **N/A:** 0 | | | **Include:** ☒ | **Exclude: ☐** | | **Seek Further Info: ☐** | | | | | | | | | | | |
| **Comments:**  This study demonstrates a high level of internal validity for a behavioral intervention by employing **concealed randomization**, **blinded outcome assessors**, and the **Intention-to-Treat (ITT)** principle, which significantly reduces selection and statistical biases,. The primary limitation is the lack of blinding for participants and interventionists, which is a common inherent challenge in technology-based clinical trials | | | | | | | | | | | | | | | | | |

## The JBI Critical Appraisal Tool for RCTs Article 7

| **Assessor:** | | | | | **Date of Appraisal:** 25 November 2025 | | **Record Number: 7** | | | | | | | | | | |
| --- | --- | --- | --- | --- | --- | --- | --- | --- | --- | --- | --- | --- | --- | --- | --- | --- | --- |
| **Study Author:** Jong-Hwan Park, Yung Liao, Du-Ri Kim, Seunghwan Song, Jun Ho Lim, Hyuntae Park, Yeanhwa Lee, dan Kyung Won Park | | | | | **Study Title:** Feasibility and Tolerability of a Culture-Based Virtual Reality (VR) Training Program in Patients with Mild Cognitive Impairment: A Randomized Controlled Pilot Study | | **Study Year:** 2020 | | | | | | | | | | |
|  | | | | |  | |  | | | | | | | | | | |
| **Internal Validity** | | | | | | **Choice - Comments/Justification** | | **Yes** | **No** | | **Unclear** | | | **N/A** | | | |
| **Bias related to selection and allocation** | | | | | | | | | | | | | | | | | |
| **1** | **Was true randomization used for assignment of participants to treatment groups?** | | | | | Participants were randomly assigned in a 1:1 ratio using SAS programming and the block randomization method*"we recruited 99 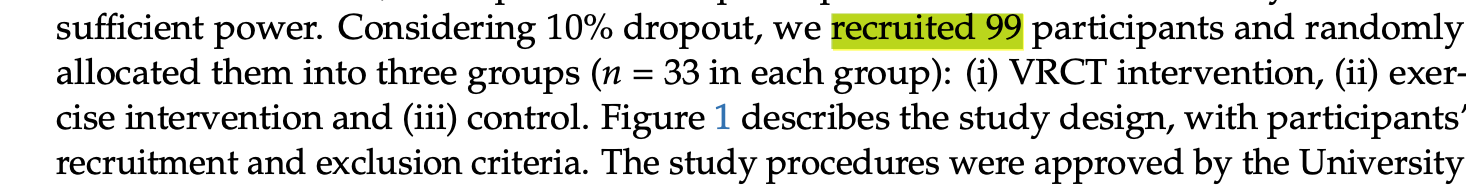* | | ☒ | ☐ | | ☐ | | | ☐ | | | |
| **2** | **Was allocation to treatment groups concealed?** | | | | | The source mentions the randomization method but does not explicitly state if the allocation was concealed from the recruiters. | | ☐ | ☐ | | ☒ | | | ☐ | | | |
| **3** | **Were treatment groups similar at the baseline?** | | | | | No significant differences were observed in socio-demographic, physical, mental, or cognitive characteristics between groups (p > 0.05)*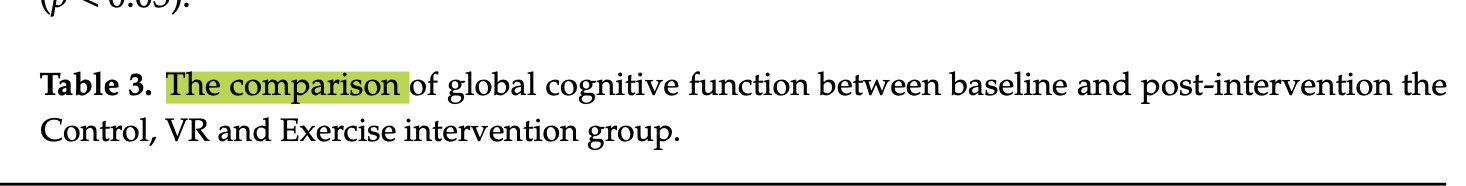* | | ☒ | ☐ | | ☐ | | | ☐ | | | |
| **Bias related to administration of intervention/exposure** | | | | | | | | | | | | | | | | | |
| **4** | **Were participants blind to treatment assignment?** | | | | | The study was a comparison between VR training and maintaining normal daily activities; blinding participants is not feasible for this design*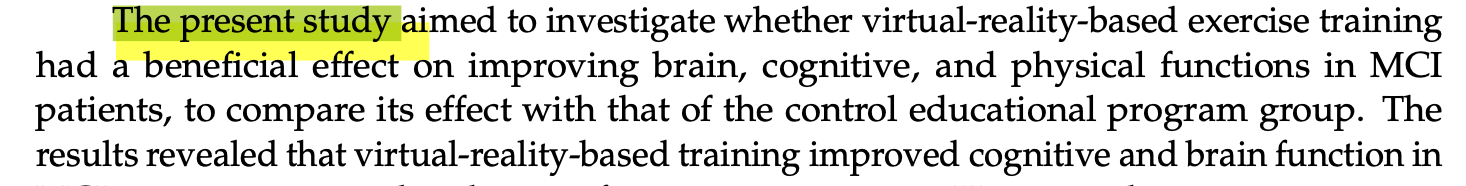* | | ☐ | ☒ | | ☐ | | | ☐ | | | |
| **5** | **Were those delivering the treatment blind to treatment assignment?** | | | | | Occupational therapists and neurologists who provided instructions and monitored the games were aware of the treatment | | ☐ | ☒ | | ☐ | | | ☐ | | | |
| **6** | **Were treatment groups treated identically other than the intervention of interest?** | | | | | The VR group received 30-minute training sessions twice a week, while the control group maintained normal lifestyles without extra stimulation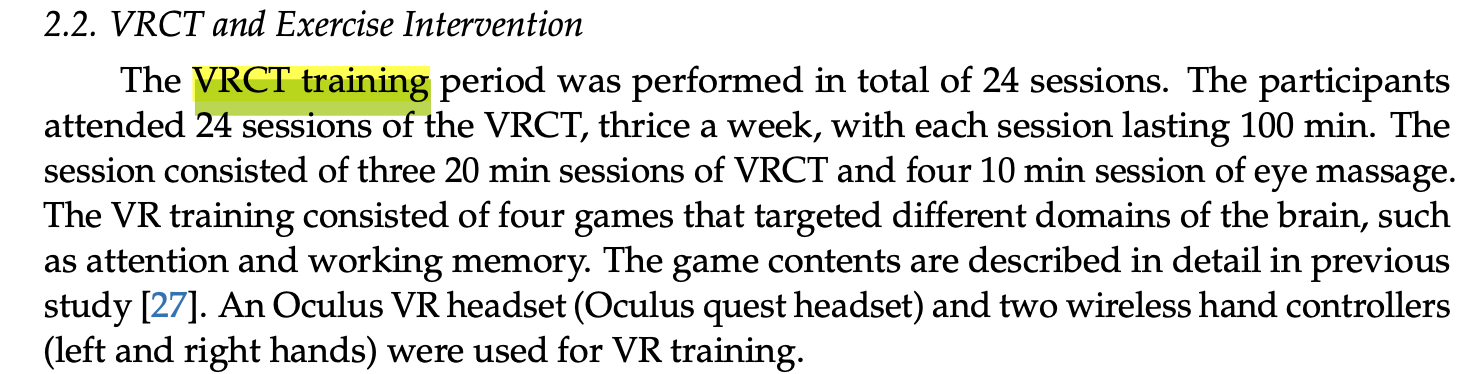 | | ☐ | ☒ | | ☐ | | | ☐ | | | |
| **Bias related to assessment, detection and measurement of the outcome** | | | | | | | | | | | | | | | | | |
| **7** | **Were outcome assessors blind to treatment assignment?** | | | | |  | | **Yes** | **No** | | **Unclear** | | | **N/A** | | | |
|  | - Outcome 1 (Global Cognition: K-MMSE) | | | | | While neuropsychologists performed the assessments, it is not stated whether they were blinded to the group assignments | | ☐ | ☐ | | ☒ | | | ☐ | | | |
|  | - Outcome 2 (Neurocognitive: Stroop/Digit Span) | | | | | There is no mention of blinded assessment for these neurocognitive tests | | ☐ | ☐ | | ☒ | | | ☐ | | | |
|  | **Outcome 3** | | | | |  | | ☐ | ☐ | | ☐ | | | ☐ | | | |
|  | **Outcome 4** | | | | |  | | ☐ | ☐ | | ☐ | | | ☐ | | | |
|  | **Outcome 5** | | | | |  | | ☐ | ☐ | | ☐ | | | ☐ | | | |
|  | **Outcome 6** | | | | |  | | ☐ | ☐ | | ☐ | | | ☐ | | | |
|  | **Outcome 7** | | | | |  | | ☐ | ☐ | | ☐ | | | ☐ | | | |
|  |  | | | | |  | |  |  | |  | | |  | | | |
| **8** | **Were outcomes measured in the same way for treatment groups?** | | | | |  | | **Yes** | **No** | | **Unclear** | | | **N/A** | | | |
|  | - - Outcome 1 (Global Cognition: K-MMSE) | | | | | Both groups were assessed at baseline and after 12 weeks using the K-MMSE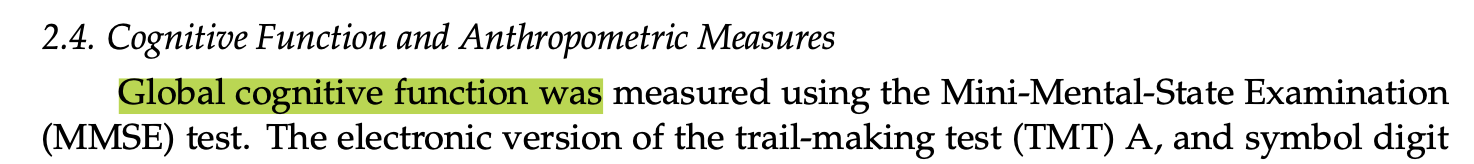 | | ☒ | ☐ | | ☐ | | | ☐ | | | |
|  | - - Outcome 2 (Neurocognitive: Stroop/Digit Span) | | | | | Both groups were assessed at baseline and after 12 weeks using the SNSB-D battery | | ☒ | ☐ | | ☐ | | | ☐ | | | |
|  | **Outcome 3** | | | | |  | | ☐ | ☐ | | ☐ | | | ☐ | | | |
|  | **Outcome 4** | | | | |  | | ☐ | ☐ | | ☐ | | | ☐ | | | |
|  | **Outcome 5** | | | | |  | | ☐ | ☐ | | ☐ | | | ☐ | | | |
|  | **Outcome 6** | | | | |  | | ☐ | ☐ | | ☐ | | | ☐ | | | |
|  | **Outcome 7** | | | | |  | | ☐ | ☐ | | ☐ | | | ☐ | | | |
|  |  | | | | |  | |  |  | |  | | |  | | | |
| **9** | **Were outcomes measured in a reliable way?** | | | | |  | | **Yes** | **No** | | **Unclear** | | | **N/A** | | | |
|  | - Outcome 1 (Global Cognition: K-MMSE) | | | | | The study used the Korean Mini-Mental State Examination (K-MMSE), a validated tool with established norms | | ☒ | ☐ | | ☐ | | | ☐ | | | |
|  | - - Outcome 2 (Neurocognitive: Stroop/Digit Span) | | | | | The tools used (Digit Span, Stroop test) were part of the validated Seoul Neuropsychological Screening Battery (SNSB-D) | | ☒ | ☐ | | ☐ | | | ☐ | | | |
|  | **Outcome 3** | | | | |  | | ☐ | ☐ | | ☐ | | | ☐ | | | |
|  | **Outcome 4** | | | | |  | | ☐ | ☐ | | ☐ | | | ☐ | | | |
|  | **Outcome 5** | | | | |  | | ☐ | ☐ | | ☐ | | | ☐ | | | |
|  | **Outcome 6** | | | | |  | | ☐ | ☐ | | ☐ | | | ☐ | | | |
|  | **Outcome 7** | | | | |  | | ☐ | ☐ | | ☐ | | | ☐ | | | |
|  |  | | | | |  | |  | | | | | | | | | |
| **Bias related to participant retention** | | | | | | | | | | | | | | | | | |
| **10** | **Was follow up complete and if not, were differences between groups in terms of their follow up adequately described and analysed?** | | | | |  | |  | | | | | | | | | |
|  | **Outcome 1:** **Primary Outcomes** | | | | |  | | **Yes** | **No** | | **Unclear** | | | **N/A** | | | |
|  |  | - Outcome 1 (Global Cognition: K-MMSE) | | | | 24 were randomized; 3 withdrew before starting (personal/medical reasons), but 100% of those who started (n=21) completed the study | | ☒ | ☐ | | ☐ | | | ☐ | | | |
|  | **Outcome 2 Secondary Outcomes** | | | | |  | | **Yes** | **No** | | **Unclear** | | | **N/A** | | | |
|  |  | - Outcome 2 (Neurocognitive: Stroop/Digit Span) | | | | All 21 participants who completed the intervention provided data for the final neurocognitive analysis | | ☒ | ☐ | | ☐ | | | ☐ | | | |
|  |  |  |  |  |  |  |  |  |  |  |  |  |  |  |  |  |  |
|  | **Statistical Conclusion Validity** | | | | | | |  |  | |  | | |  | | | |
| **11** | **Were participants analysed in the groups to which they were randomized?** | | | | |  | |  | | | | | | | | | |
|  | **Outcome 1:** **Primary Outcomes** | | | | |  | | **Yes** | **No** | | **Unclear** | | | **N/A** | | | |
|  |  | - Outcome 1 (Global Cognition: K-MMSE) | | | | The analysis was "per-protocol" based on the 21 participants who received the intervention, excluding the 3 who were randomized but did not start | | ☐ | ☒ | | ☐ | | | ☐ | | | |
|  | **Outcome 2 Second Outcomes** | | | | |  | | **Yes** | **No** | | **Unclear** | | | **N/A** | | | |
|  |  | - Outcome 2 (Neurocognitive: Stroop/Digit Span) | | | | Similar to Outcome 1, analysis included only the final 21 participants | | ☐ | ☒ | | ☐ | | | ☐ | | | |
|  |  |  |  |  |  |  |  |  |  |  |  |  |  |  |  |  |  |
|  |  | | | | | | |  |  | | | |  | |  | | |
| **12** | **Was appropriate statistical analysis used?** | | | | |  | |  |  | | | |  | |  | | |
|  | **Outcome 1:** **Primary Outcomes** | | | | |  | | **Yes** | **No** | | | | **Unclear** | | **N/A** | | |
|  |  | - Outcome 1 (Global Cognition: K-MMSE) | | | | Normality was checked (Shapiro–Wilk), and repeated-measures ANOVA was used to determine interaction effects | | ☒ | ☐ | | | | ☐ | | ☐ | | |
|  | **Outcome 2 Second Outcomes** | | | | |  | | **Yes** | **No** | | | **Unclear** | | | | **N/A** | |
|  |  | - Outcome 2 (Neurocognitive: Stroop/Digit Span) | | | | Repeated-measures ANOVA was used for interactions, and Student's t-test compared baseline variables | | ☒ | ☐ | | | ☐ | | | | ☐ | |
|  |  | | | | |  | |  | |  | |  | | | | |  |
|  |  | | | | |  | | **Yes** | | **No** | | **Unclear** | | | | | **N/A** |
| **13** | **Was the trial design appropriate and any deviations from the standard RCT design (individual randomization, parallel groups) accounted for in the conduct and analysis of the trial?** | | | | | The study used a randomized controlled pilot design, which is appropriate for examining feasibility and tolerability | | ☒ | | ☐ | | ☐ | | | | | ☐ |
| **Overall appraisal:**  • **Yes:** 12  • **No:** 5  • **Unclear:** 3  • **N/A:** 0 | | | **Include:** ☒ | **Exclude: ☐** | | **Seek Further Info: ☐** | | | | | | | | | | | |
| **Comments:**  The study by **Park J-H et al. (2020)** exhibits strong selection validity through **true randomization (SAS-based)** and **baseline similarity** of the groups. Its primary limitations are the **unblinded design** (open-label) and **per-protocol analysis**, as three participants were excluded from the final analysis after randomization occurred. However, for a pilot study focused on feasibility, the use of **standardized and reliable measurement tools** (SNSB-D and K-MMSE) maintains a high level of data quality | | | | | | | | | | | | | | | | | |

## The JBI Critical Appraisal Tool for RCTs Article 8

| **Assessor:** | | | | | **Date of Appraisal:** 30 November 2025 | | **Record Number: 8** | | | | | | | | | | |
| --- | --- | --- | --- | --- | --- | --- | --- | --- | --- | --- | --- | --- | --- | --- | --- | --- | --- |
| **Study Author:**  Ji-Su Park, Young-Jin Jung, dan Gihyoun Lee | | | | | **Study Title:**  Virtual Reality-Based Cognitive–Motor Rehabilitation in Older Adults with Mild Cognitive Impairment: A Randomized Controlled Study on Motivation and Cognitive Function | | **Study Year:** 2020 | | | | | | | | | | |
|  | | | | |  | |  | | | | | | | | | | |
| **Internal Validity** | | | | | | **Choice - Comments/Justification** | | **Yes** | **No** | | **Unclear** | | | **N/A** | | | |
| **Bias related to selection and allocation** | | | | | | | | | | | | | | | | | |
| **1** | **Was true randomization used for assignment of participants to treatment groups?** | | | | | Participants were randomly assigned to the VRCMR or CCR group using **blocked randomization**  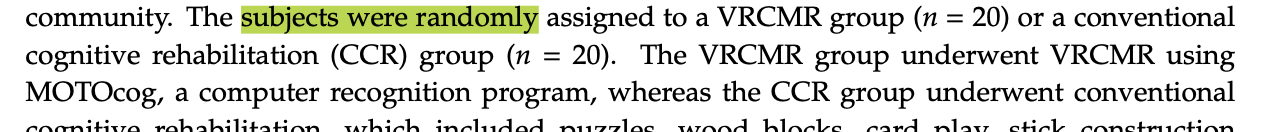 | | ☒ | ☐ | | ☐ | | | ☐ | | | |
| **2** | **Was allocation to treatment groups concealed?** | | | | | Allocation was concealed using **sealed opaque envelopes**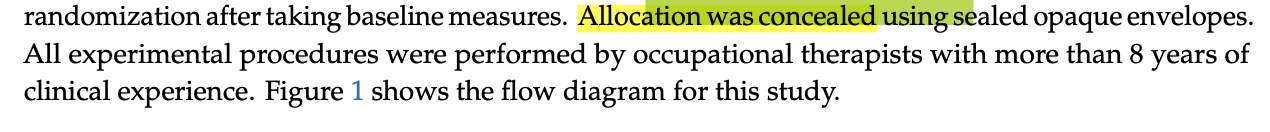 | | ☒ | ☐ | | ☐ | | | ☐ | | | |
| **3** | **Were treatment groups similar at the baseline?** | | | | | There were no significant differences between groups regarding general characteristics or baseline cognitive scores (MoCA, TMT, DST) | | ☒ | ☐ | | ☐ | | | ☐ | | | |
| **Bias related to administration of intervention/exposure** | | | | | | | | | | | | | | | | | |
| **4** | **Were participants blind to treatment assignment?** | | | | | Due to the distinct nature of VR equipment versus tabletop activities (puzzles/blocks), participants were aware of their group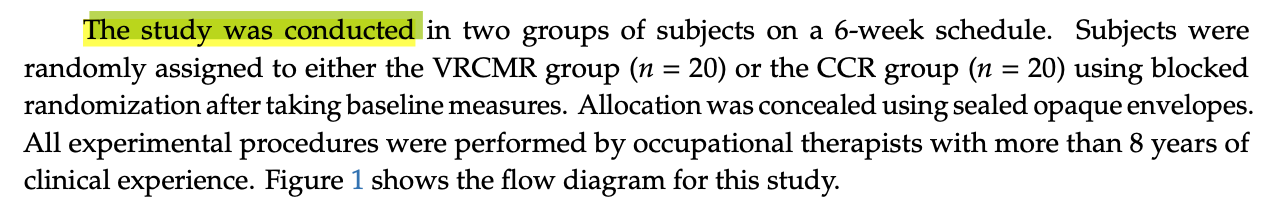 | | ☐ | ☒ | | ☐ | | | ☐ | | | |
| **5** | **Were those delivering the treatment blind to treatment assignment?** | | | | | Sessions were conducted by occupational therapists who directly managed the specific VR software or CCR materials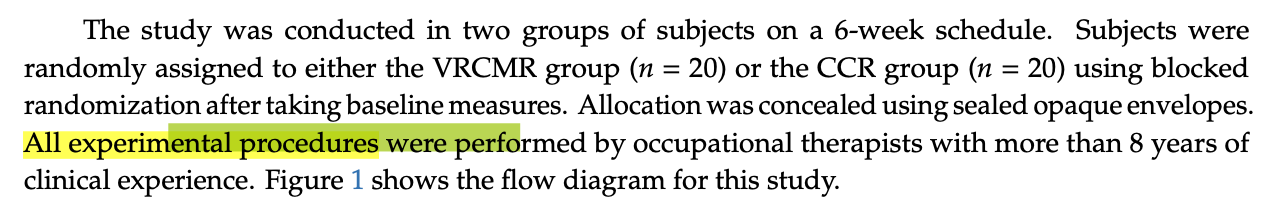 | | ☐ | ☒ | | ☐ | | | ☐ | | | |
| **6** | **Were treatment groups treated identically other than the intervention of interest?** | | | | | Both groups received intervention for the **same duration (30 min/day)** and **frequency (5 days/week for 6 weeks)**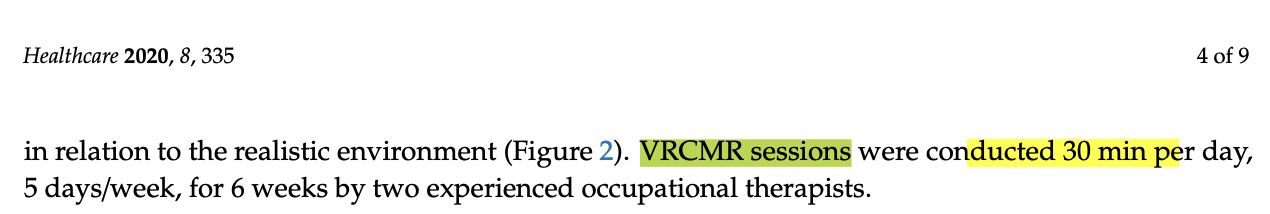 | | ☒ | ☐ | | ☐ | | | ☐ | | | |
| **Bias related to assessment, detection and measurement of the outcome** | | | | | | | | | | | | | | | | | |
| **7** | **Were outcome assessors blind to treatment assignment?** | | | | |  | | **Yes** | **No** | | **Unclear** | | | **N/A** | | | |
|  | - Outcome 1 (MoCA/Cognition) | | | | | The source does not explicitly state if the assessors were blinded to the group assignments during post-training evaluations | | ☐ | ☐ | | ☒ | | | ☐ | | | |
|  | - Outcome 2 (TMT/DST/Motivation) | | | | | No specific mention of assessor blinding for these secondary outcomes | | ☐ | ☐ | | ☒ | | | ☐ | | | |
|  |  | | | | |  | |  |  | |  | | |  | | | |
| **8** | **Were outcomes measured in the same way for treatment groups?** | | | | |  | | **Yes** | **No** | | **Unclear** | | | **N/A** | | | |
|  | - Outcome 1 (MoCA/Cognition) | | | | | Both groups were evaluated using the same MoCA scale before and after the 6-week intervention | | ☒ | ☐ | | ☐ | | | ☐ | | | |
|  | - Outcome 2 (TMT/DST/Motivation) | | | | | Both groups used the same TMT, DST, and NRSS motivation scales | | ☒ | ☐ | | ☐ | | | ☐ | | | |
|  |  | | | | |  | |  |  | |  | | |  | | | |
| **9** | **Were outcomes measured in a reliable way** | | | | |  | | **Yes** | **No** | | **Unclear** | | | **N/A** | | | |
|  | - Outcome 1 (MoCA/Cognition) | | | | | The study utilized tests with **proven sensitivity, validity, and reliability**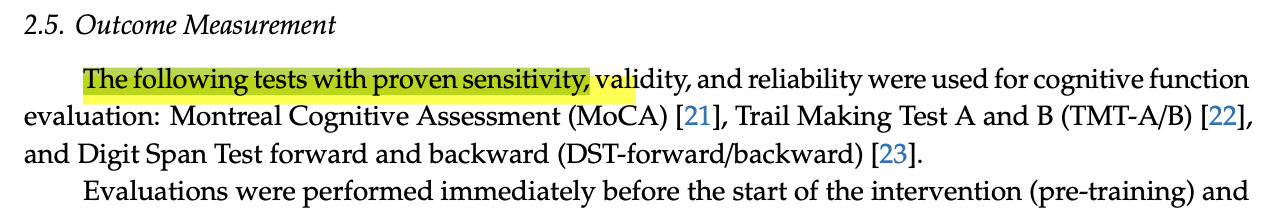 | | ☒ | ☐ | | ☐ | | | ☐ | | | |
|  | - Outcome 2 (TMT/DST/Motivation) | | | | | These are standardized tools; motivation was measured via a recognized numeric rating self-report scale (NRSS)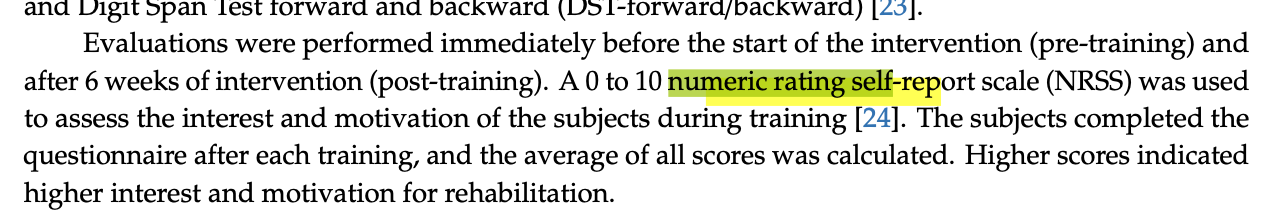 | | ☐ | ☐ | | ☒ | | | ☐ | | | |
|  |  | | | | |  | |  | | | | | | | | | |
| **Bias related to participant retention** | | | | | | | | | | | | | | | | | |
| **10** | **Was follow up complete and if not, were differences between groups in terms of their follow up adequately described and analysed?** | | | | |  | |  | | | | | | | | | |
|  | **Outcome 1:** **Primary Outcomes** | | | | |  | | **Yes** | **No** | | **Unclear** | | | **N/A** | | | |
|  |  | - Outcome 1 (MoCA/Cognition) | | | | 5 subjects dropped out (2 VR, 3 CCR). The reasons (refusal or poor participation) were explicitly described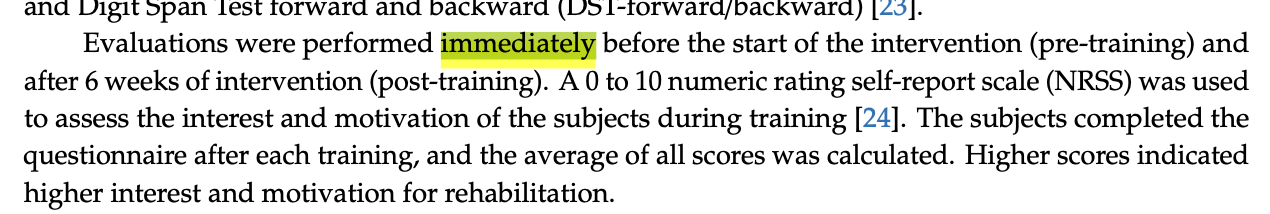 | | ☒ | ☐ | | ☐ | | | ☐ | | | |
|  | **Outcome 2 Second Outcomes** | | | | |  | | **Yes** | **No** | | **Unclear** | | | **N/A** | | | |
|  |  | - Outcome 2 (TMT/DST/Motivation) | | | | Dropout rates and reasons were consistently reported for all outcome measures | | ☒ | ☐ | | ☐ | | | ☐ | | | |
|  | **Statistical Conclusion Validity** | | | | | | |  |  | |  | | |  | | | |
| **11** | **Were participants analysed in the groups to which they were randomized?** | | | | |  | |  | | | | | | | | | |
|  | **Outcome 1:** **Primary Outcomes** | | | | |  | | **Yes** | **No** | | **Unclear** | | | **N/A** | | | |
|  |  | - Outcome 1 (MoCA/Cognition) | | | | The analysis was conducted only on the **35 subjects who completed the study**, indicating a per-protocol rather than an intention-to-treat (ITT) analysis | | ☐ | ☒ | | ☐ | | |  | | | |
|  | **Outcome 2 Second Outcomes** | | | | |  | | **Yes** | **No** | | **Unclear** | | | **N/A** | | | |
|  |  | - Outcome 2 (TMT/DST/Motivation) | | | | Only the 35 subjects who finished the study were included in the final analysis | | ☐ | ☒ | | ☐ | | |  | | | |
| **12** | **Was appropriate statistical analysis used?** | | | | |  | |  |  | | | |  | |  | | |
|  | **Outcome 1:** Primary Outcomes | | | | |  | | **Yes** | **No** | | | | **Unclear** | | **N/A** | | |
|  |  | - Outcome 1 (MoCA/Cognition) | | | | Shapiro-Wilk test checked normality; paired and independent t-tests were used appropriately for comparisons | | ☒ | ☐ | | | | ☐ | | ☐ | | |
|  | **Outcome 2** Second Outcomes | | | | |  | | **Yes** | **No** | | | **Unclear** | | | | **N/A** | |
|  |  | - Outcome 2 (TMT/DST/Motivation) | | | | Effect sizes (Cohen’s d) were calculated to determine the magnitude of changes | | ☒ | ☐ | | | ☐ | | | | ☐ | |
|  |  | | | | |  | |  | |  | |  | | | | |  |
|  |  | | | | |  | | **Yes** | | **No** | | **Unclear** | | | | | **N/A** |
| **13** | **Was the trial design appropriate and any deviations from the standard RCT design (individual randomization, parallel groups) accounted for in the conduct and analysis of the trial?** | | | | | The study used a randomized controlled trial (RCT) design, which is the standard for testing intervention efficacy | | ☒ | | ☐ | | ☐ | | | | | ☐ |
| **Overall appraisal:**  • **Yes:** 13  • **No:** 4  • **Unclear:** 2  • **N/A:** 0 | | | **Include:** ☒ | **Exclude: ☐** | | **Seek Further Info: ☐** | | | | | | | | | | | |
| **Comments:**  The study by **Park J-S et al. (2020)** demonstrates strong validity in its **randomization process** and **baseline comparability**. It successfully controlled for "time on task" by ensuring both groups received identical amounts of therapy. However, its main limitations for internal validity are the **lack of blinding** (common in physical/cognitive rehab studies) and the **exclusion of dropouts** from the final statistical analysis, which prevents it from being a true Intention-To-Treat study | | | | | | | | | | | | | | | | | |
